# Amorphous K‐Buserite Microspheres for High‐Performance Aqueous Zn‐Ion Batteries and Hybrid Supercapacitors

**DOI:** 10.1002/advs.202207329

**Published:** 2023-02-24

**Authors:** Zhi‐Qiang Wang, Hong‐Ming Chen, Xiao‐Dong Liu, Li‐Ying Song, Bu‐Sheng Zhang, Yun‐Guo Yang, Zhao‐Cheng Zhang, Qian Li, Tian‐Qi Gao, Jing Bai, Woon‐Ming Lau, Dan Zhou

**Affiliations:** ^1^ Beijing Advanced Innovation Center for Materials Genome Engineering and Center for Green Innovation School of Mathematics and Physics University of Science and Technology Beijing Beijing 100083 P. R. China; ^2^ Shunde Innovation School University of Science and Technology Beijing Foshan Guangdong 528000 P. R. China; ^3^ Center for Electron Microscopy and Tianjin Key Laboratory of Advanced Functional Porous Materials Institute for New Energy Materials and Low‐Carbon Technologies School of Materials Science and Engineering Tianjin University of Technology Tianjin 300384 P. R. China; ^4^ The Center of New Energy Materials and Technology School of Materials Science and Engineering Southwest Petroleum University Chengdu Sichuan 610500 P. R. China

**Keywords:** amorphous K‐buserite, aqueous Zn‐ion batteries, aqueous Zn‐ion hybrid supercapacitors, cathode, Zn‐buserite

## Abstract

Aqueous Zn‐ion batteries (AZIBs) and Zn‐ion hybrid supercapacitors (AZHSCs) are considered promising energy‐storage alternatives to Li‐ion batteries due to the attractive merits of low‐price and high‐safety. However, the lack of suitable cathode materials always hinders their large‐scale application. Herein, amorphous K‐buserite microspheres (denoted as K‐MnO*_x_*) are reported as cathode materials for both AZIBs and AZHSCs, and the energy‐storage mechanism is systematically revealed. It is found that K‐MnO*_x_* is composed of rich amorphous K‐buserite units, which can irreversibly be transformed into amorphous Zn‐buserite units in the first discharge cycle. Innovatively, the transformed Zn‐buserite acts as active materials in the following cycles and is highly active/stable for fast Zn‐diffusion and superhigh pseudocapacitance, enabling the achievement of high‐efficiency energy storage. In the AZIBs, K‐MnO*_x_* delivers 306 mAh g^−1^ after 100 cycles at 0.1 A g^−1^ with 102% capacity retention, while in the AZHSCs, it shows 515.0/116.0 F g^−1^ at 0.15/20.0 A g^−1^ with 92.9% capacitance retention at 5.0 A g^−1^ after 20 000 cycles. Besides, the power/energy density of AZHSCs device can reach up to 16.94 kW kg^−1^ (at 20 A g^−1^)/206.7 Wh kg^−1^ (at 0.15 A g^−1^). This work may provide some references for designing next‐generation aqueous energy‐storage devices with high energy/power density.

## Graphical abstract

Herein, an amorphous K‐buserite microspheres (K‐MnO*_x_*) cathode material is developed for aqueous Zn‐ion batteries and Zn‐ion hybrid supercapacitors. The K‐MnO*_x_* is composed of numerous amorphous K‐buserite units, which are irreversibly transformed into amorphous Zn‐buserite units in the first discharge cycle. The Zn‐buserite is highly active/stable for high‐efficiency energy‐storage.

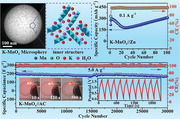

## 1 Introduction

The development of high‐performance energy‐storage devices is currently urgent and imperative to solve the issues of increasing environmental pollution and energy deficiency.^[^ ^1^, ^2^, ^3^, ^4^, ^5^, ^6^ ^]^ Till now, several energy‐storage devices, such as Li‐ion batteries (LIBs),^[^ ^7^ ^]^ lead‐acid batteries,^[^ ^8^ ^]^ supercapacitors,^[^ ^5^ ^]^ alkaline zinc batteries,^[^ ^9^ ^]^ have been widely explored. However, some intrinsic defects inevitably hinder their wide application in large‐scale energy‐storage equipment (e.g., portable electronics and electric vehicles). For example, LIBs deliver fairly low power density and worrisome safety issues; Lead‐acid batteries and supercapacitors are reported to possess a relatively low energy density. As for alkaline zinc/manganese oxide batteries, they commonly exhibit poor cycling stability.

Combining the advantages of the high energy density of battery‐type electrodes and the high power density of capacitor‐type electrodes, varieties of hybrid supercapacitors (HSCs) are proposed recently, including monovalent (Li^+^, Na^+^, and K^+^) and multivalent (Ca^2+^, Zn^2+^, and Al^3+^) HSCs.^[^ ^2^, ^3^, ^5^ ^]^ Among them, aqueous Zn‐ion hybrid supercapacitors (AZHSCs) have received wide attention due to the integrated advantages of aqueous zinc‐ion batteries (AZIBs) and supercapacitors, such as high theoretical capacities, high rate capabilities, cheap prices, great safety, and environmental friendliness. In recent years, various activated carbon (AC) materials have been widely reported as AZHSCs cathode materials with a Zn metal plate as the anode for each AC//Zn device.^[^ ^10^, ^11^, ^12^ ^]^ These reports have shown that AC is very stable. Nevertheless, the severe growth of zinc dendrites on the anode during charging is a key issue in an AZHSCs system, causing the devices to short‐circuit and have poor cycling stability.^[^ ^13^, ^14^ ^]^ An effective strategy to overcome this challenge is replacing the metallic Zn anode with other anode materials. After referencing Guo's^[^ ^15^ ^]^ and Zhang's^[^ ^16^ ^]^ works, it was found that AC can be a good substitute material for Zn metal anode in the AZHSCs devices due to its brilliant cycling stability in aqueous Zn‐ion systems.

However, AC acts as the cathode material in a common AC//Zn AZHSCs device. When using AC as an anode material instead of Zn anode, a cathode material with high working voltage is needed, which is in favor of high working voltage and specific capacitance of the device. Among various reported AZIBs cathode materials, including manganese‐based materials,^[^ ^17^, ^18^, ^19^, ^20^ ^]^ vanadium‐based candidates,^[^ ^21^, ^22^, ^23^, ^24^, ^25^, ^26^ ^]^ and Prussian blue analogs,^[^ ^27^, ^28^ ^]^ Mn‐based materials demonstrate the highest operating voltage (≈1.3 V vs Zn/Zn^2+^) and suggest a promising potential as cathode materials for AZHSCs. However, the Mn‐based materials always deliver poor cycling/rate performance due to the large radius and strong electrostatic interaction of bivalent Zn^2+^.^[^ ^13^, ^29^, ^30^ ^]^ To address these problems, Srinivasan and co‐workers^[^ ^31^ ^]^ reported an amorphous manganese dioxide (A‐MnO_2‐*δ*_) instead of crystalline MnO_2_ polymorphs as a cathode material of AZIBs. The A‐MnO_2‐*δ*_ has rich structural defects, inherent isotropic nature as well as better reaction kinetics than its crystalline counterparts, hence exhibiting a high specific capacity, great rate capability, and long cycling life. In addition, buserite manganese oxides (K‐, ^[^ ^32^, ^33^ ^]^ Mg‐, ^[^ ^34^ ^]^ Zn‐^[^ ^35^ ^]^ buserite, etc.) are also reported as suitable cathode materials due to their unique layer structure with 0.7 nm interplane space. Inspired by these virtues of amorphous nature and buserite phase, an amorphous K‐buserite was designed and prepared as the cathode material for AZIBs and AZHSCs.

Herein, we report amorphous K‐buserite microspheres (denoted as K‐MnO*_x_*) as the cathode material of AZIBs and AZHSCs. First, the fabrication process, composition information, and intrinsic structures of K‐MnO*_x_* were described. Afterward, the energy‐storage mechanism was revealed by a series of ex situ observations (including X‐ray photoelectron spectroscopy (XPS), X‐ray diffraction (XRD), Raman, scanning electron microscopy (SEM), and transmission electron microscopy (TEM)) combined with the electrochemical behaviors. It is found that the amorphous K‐buserite is fully transformed into amorphous Zn‐buserite in the first discharge process, which then participates in the subsequent electrochemical reaction and acts as the host material in AZIBs and AZHSCs. It is also confirmed that the transformation of Zn‐buserite from K‐buserite is irreversible, and in other words, the Zn‐buserite cannot be transformed back into K‐buserite during the following charge process. The transformed Zn‐buserite possesses the virtues of amorphous MnO*_x_* and crystalline K‐buserite phases, including low charge‐transfer resistance, enhanced Zn diffusion rate, and high pseudocapacitance contribution, and therefore it delivers desired energy‐storage performance in both AZIBs and AZHSCs devices. As a result, in the AZIBs device (K‐MnO*_x_*//Zn), K‐MnO*_x_* delivers a specific capacity of 306.6 mAh g^−1^ at 0.1 A g^−1^ after 100 cycles with 102% capacity retention, along with good cycling performance and rate capability, such as remaining 60 mAh g^−1^ after 3000 cycles at 1.0 A g^−1^. In the AZHSCs device (K‐MnO*_x_*//AC), the K‐MnO*_x_* shows a specific capacitance of 515.0/297.8/194.1/116.0 F g^−1^ at 0.15/2.0/10.0/20.0 A g^−1^, and it can maintain 92.9%/81.8% specific capacitance at 5.0 A g^−1^ after 20 000/30 000 cycles. This work might provide a new option for the construction of high‐performance AZIBs and AZHSCs devices.

## 2 Results and Discussions

**Figure 1:**
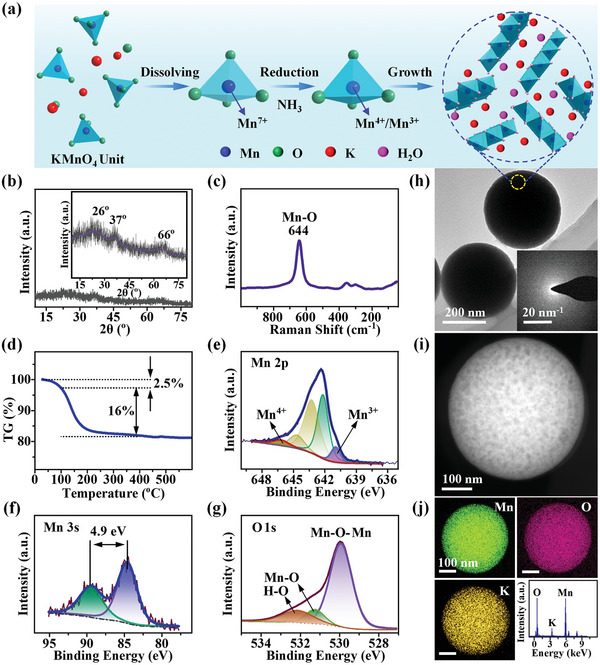
a) Schematical formation mechanism and b–j) physicochemical characteristics of K‐MnO*_x_*. (b) XRD pattern and the inset is an enlarged figure of the XRD pattern (the blue dot line is a smoothed result of the black pattern), (c) Raman spectrum, (d) TGA curve, (e) Mn 2p, (f) Mn 3s, and (g) O 1s XPS spectra. (h) TEM image and the inset is the SAED pattern of a single microsphere. (i) HADDF‐STEM image. (j) TEM‐mapping images and EDS spectrum.

The preparation process of K‐MnO*_x_* microspheres is schematically illustrated in **Figure** **1**a. In the first step, KMnO_4_ was dissolved in a concentrated ammonia solution (≈28%, NH_3_ H_2_O). Subsequently, the dissolved Mn^7+^ in MnO_4_^−^ is reduced into Mn^4+^ and Mn^3+^ by ammonia, which can be confirmed by the change of the solution/sample colors (Figure S1a,b, Supporting Information). The color of the newly prepared KMnO_4_ solution is purple (the characteristic color of Mn^7+^ in MnO_4_^−^), and it changes to brown quickly with the ammonia reduction of Mn^7+^. After that, Mn^4+^ and Mn^3+^ coprecipitate with K atoms and water molecules in concentrated NH_3_ H_2_O solution to form the K‐MnO*_x_* microspheres. During this process, manganese oxide octahedral species formed first, which then rapidly self‐assembled via the Mn–O–Mn networks, forming amorphous K‐MnO*_x_* clusters gradually. Finally, the K‐MnO*_x_* sample was collected by filtering and drying.^[^ ^36^ ^]^ This preparation process is easy and promising for large‐scale production.

The crystalline structure of K‐MnO*_x_* was investigated by XRD, as shown in Figure 1b. The XRD pattern does not display any intense diffraction peak, suggesting that K‐MnO*_x_* is amorphous. However, three weak peaks at 26°, 37°, and 66° can be observed in the enlarged XRD pattern (the inset of Figure 1b; the blue dot line smoothed from the XRD pattern shows the changing trend of it), indicating that K‐MnO*_x_* includes rich mid/short‐range order structure units of MnO_6_ octahedral frameworks.^[^ ^15^, ^36^ ^]^ It is these mid/short‐range‐order structure units that lead to these broad peaks.^[^ ^15^, ^32^ ^]^ The peak position of the three well agrees with that of (002), (−111), and (311) diffraction peaks of K‐buserite (JCPDS #80‐1098), respectively.^[^ ^32^, ^37^ ^]^ Therefore, it is concluded that K‐MnO*_x_* is mainly composed of mid/short‐range order K‐buserite units, and its inner structure can be schematically exhibited in the right of Figure 1a. Furthermore, the Raman spectrum in Figure 1c displays a sharp peak located at 644 cm^−1^, which is associated with the characteristic Mn–O stretching vibration of MnO_6_ structural units, agreeing well with the short‐range‐order MnO_6_ frameworks of K‐buserite.^[^ ^15^, ^36^ ^]^ K‐buserite commonly contains crystal water and water molecules in its crystalline structure to keep its structural stability.^[^ ^34^ ^]^ The water content in K‐MnO*_x_* was analyzed by thermogravimetric analyses (TGA) in Figure 1d. The TGA curve shows that 18.5 wt% water includes in K‐MnO*_x_*, in which about 16 wt% is attributed to the crystal water removed above 100 °C, while the other 2.5% is due to the physically adsorbed water evaporated below 100 °C.

XPS spectra were obtained to analyze the compositional information and chemical state of K‐MnO*_x_*. The survey spectrum (Figure S1c, Supporting Information) displays strong signals of Mn, O, K, and C without impure peaks. The atomic ratio of K, Mn, and O is calculated to be 0.186:1:1.387, according to the XPS results. Figure 1e demonstrates the Mn 2p spectrum with a spin energy separation of 11.61 eV, revealing that Mn^4+^ and Mn^3+^ are the dominant oxidation states of Mn in K‐MnO*_x_*.^[^ ^15^, ^38^ ^]^ Based on Biesinger's fitting method,^[^ ^38^ ^]^ the Mn 2p_3/2_ peak can be fitted into five peaks at 640.97, 652.16, 643.23, 644.64, and 646.19 eV. Among them, the peaks at 640.97 and 646.19 eV are attributed to Mn^3+^ and Mn^4+^, respectively, while the other three peaks are the satellite peaks of Mn at oxidation states.^[^ ^38^ ^]^ The Mn 3s spectrum (Figure 1f) displays a multiple energy splitting of 4.9 eV (between 5.4 eV for Mn_2_O_3_ and 4.8 eV for MnO_2_), which also indicates the coexistence of Mn^3+^ and Mn^4+^ in the sample. The average oxidation state of Mn is calculated to be 3.38 according to the magnitude of energy splitting,^[^ ^36^, ^39^ ^]^ corresponding to the mixed valence state of Mn 2p. The asymmetrical O 1s spectrum of K‐MnO*_x_* (Figure 1g) is composed of three peaks at 529.93, 531.25, and 532.14 eV. Among them, the peaks at 529.93 and 531.25 eV are attributed to the lattice oxygen of MnO_6_ and the hydroxyl groups of defective O,^[^ ^31^, ^40^ ^]^ respectively, and the peak at 532.14 eV is assigned to the O in crystal water.^[^ ^15^ ^]^ Moreover, Figure S1d of the Supporting Information shows the K 2p spectra of K‐MnO*_x_* and crystalline KOH. The K 2p binding energy of K‐MnO*_x_* at 292.62 eV is much lower than that of KOH at 293.78 eV, indicating that K atoms exist in K‐MnO*_x_* via the relatively weak interaction.

The morphology and structure of K‐MnO*_x_* were investigated by SEM and TEM‐energy dispersive spectroscopy (EDS)‐selected area electron diffraction (SAED)‐Mapping tests. Figure 1h and Figure S1e (Supporting Information) display the SEM and TEM‐SAED images of K‐MnO*_x_*. As shown, the K‐MnO*_x_* includes many monodispersed microspheres with an average diameter of 300–500 nm, which agrees with its particle size distribution (the left‐bottom inset of Figure S1e, Supporting Information). The SAED pattern (the inset of Figure 1h) does not demonstrate any clear light spot or diffraction cycle, suggesting that the K‐MnO*_x_* is amorphous, which is consistent with the above XRD result. The high‐angle annular dark field‐scanning transmission electron microscopy (HAADF‐STEM) image in Figure 1i reveals some inner structural information about K‐MnO*_x_*. There are numerous structural defects and nanopores due to that the K‐MnO*_x_* is composed of mid/short‐range‐order K‐buserite units. The specific surface area and pore diameter distribution of the K‐MnO*_x_* were further investigated by an N_2_ adsorption–desorption test. The adsorption–desorption isotherms in Figure S1g of the Supporting Information display a type‐II branch with an H_3_ hysteresis loop, indicating some inner pore structure in the K‐MnO*_x_* sample. The *S*_BET_ of K‐MnO*_x_* is 153.6 m^2^ g^−1^ estimated from the isotherms. The large *S*_BET_ value indicates numerous active sites for Zn‐storage in K‐MnO*_x_*. Its pore size distribution information is in Figure S1h of the Supporting Information. The figure shows both peaks, and the right one (20–200 nm) is attributed to the K‐MnO*_x_*‐nanosphere‐accumulation pores, while the left one between 2 and 5 nm is due to the inner mesopores in K‐MnO*_x_* spheres, confirming the above HAADF‐STEM observation. The pore structure enables fast Zn diffusion, contributing to the efficient Zn‐storage in this material. The Mapping‐EDS images in Figure 1j show the coexistence of K, Mn, and O elements, and the corresponding atomic percentages are estimated to be 7.5%, 40.6%, and 51.9%, respectively. The ratio of K:Mn is 1:1.54, which is in good accordance with the XPS and ICP‐MS results, as shown in Table S1 of the Supporting Information. According to the above TEM–XPS–TGA analyses, the chemical formula of K‐MnO*_x_* is approximately inferred to be K_0.185_MnO_3.88_·0.16H_2_O. After an annealing treatment at 500 °C for 2 h, the morphology of K‐MnO*_x_* remained still, whereas its crystal water is removed and K‐buserite is transformed into the K‐MnO_2_. All these can be confirmed by the XRD, XPS, SEM, and TEM‐Mapping results of K‐MnO_2_, as shown in Figure S2a–e of the Supporting Information.

**Figure 2:**
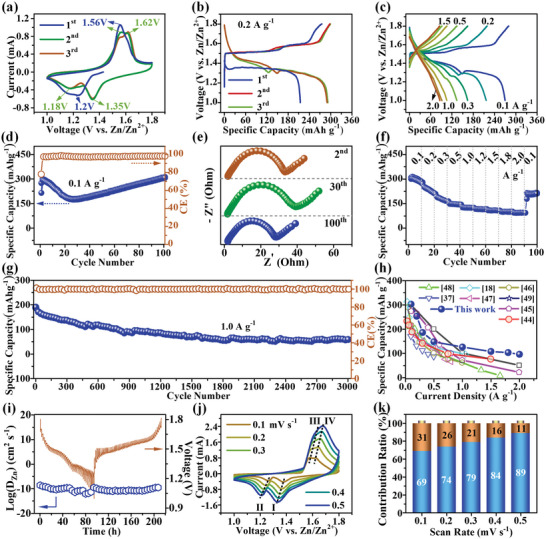
Electrochemical performance of K‐MnO*_x_* in K‐MnO*_x_*//Zn. a) CV curves (at 0.2 mV s^−1^). b) GCD curves (at 0.2 A g^−1^). c) Comparison of GCD curves acquired at various current densities (0.1–2.0 A g^−1^). d) Cycling performance at 0.1 A g^−1^ and the corresponding coulombic efficiency (CE). e) EIS plots of K‐MnO*_x_* electrode tested after the 2nd, 30th, and 100th cycles. f) Rate properties at various current densities. g) Long‐cycling performance at 1.0 A g^−1^. h) Comparison of Zn‐storage performance with the previously reported Mn‐based cathode materials, including MnO_2_,^[^ ^44^ ^]^ MnO_2_/MWCNT,^[^ ^45^ ^]^ layered MnO_2_,^[^ ^46^ ^]^ a‐MnO*_x_*,^[^ ^47^ ^]^ *δ*‐MnO_2_,^[^ ^48^ ^]^ *β*‐MnO_2_ nanorod,^[^ ^18^ ^]^ *δ*‐MnO_2_ NSs,^[^ ^37^ ^]^ and IO‐MnO_2_. ^[^ ^49^ ^]^ i) GITT curves and corresponding Zn^2+^ diffusion coefficient acquired at a GCD current of 0.05 mA. j) CV curves at scan rates from 0.1 to 0.5 mV s^−1^. k) Calculated pseudocapacitance contribution percentage at each scan rate (0.1–0.5 mV s^−1^).

The electrochemical properties of K‐MnO*_x_* as an AZIBs cathode were examined by cyclic voltammogram (CV) (**Figure** **2**a) and galvanostatic charge–discharge (GCD) (Figure 2b) curves. In the first discharge process, a broad peak at 1.2 V is observed in the CV curve, and a plateau between 1.4 and 1.25 V in the GCD profile, both of which are attributed to the coinsertion of hydrogen protons (H^+^) and Zn‐ions (Zn^2+^) into amorphous K‐MnO*_x_*.^[^ ^41^, ^42^ ^]^ During the following charge cycle, a strong peak at 1.56 V appears along with a short shoulder peak (at ≈1.62 V), which can be ascribed to the extraction of Zn^2+^ and H^+^, respectively. In the second cycle, the CV curve exhibits four peaks at 1.35, 1.18, 1.56, and 1.62 V. The peak at 1.35/1.62 V is mainly due to the insertion/extraction of H^+^, and the peak at 1.18/1.56 V is chiefly attributed to the insertion/extraction of Zn^2+^. The capacity‐contribution percentage of protons to the total specific capacity of K‐MnO*_x_* can be roughly evaluated according to the GCD curves acquired at various current densities. As seen in Figure 2c (between 1.3 and 1.8 V in discharge curves), the percentage increases gradually with the rising of current densities from 0.1 to 2.0 A g^−1^, and the estimated percentage at each current density is displayed in Figure S3a,b of the Supporting Information. As seen, when a small current density of 0.1 A g^−1^ is used, the capacity‐contribution percentage of H^+^ is ≈55%, and it increases significantly to 79% at 2.0 A g^−1^. Therefore, H^+^ plays an important role in the total capacity of K‐MnO*_x_*.

The cycling performance of K‐MnO*_x_* is shown in Figure 2d. The first discharge/charge specific capacity is 214.4/275.2 mAh g^−1^ at 0.1 A g^−1^ with an initial coulombic efficiency (CE) of 78%. In the 2nd cycle, the specific capacity rises to 300 mAh g^−1^, and the CE reaches up to near 100%. During the initial 27 cycles, the specific capacity decreases gradually to 178 mAh g^−1^, and then it increases steadily to 306.6 mAh g^−1^ at the 100th cycle with a capacity retention ratio of 102% in comparison with that of the 2nd cycle. The incipient decrease and then increase of the specific capacity is due to the gradual activation of the K‐MnO*_x_* electrode, confirmed by CV curves (Figure 2a; Figure S3c, Supporting Information) and GCD profiles (Figure S3d,e, Supporting Information). More detailedly, the shape of the CV curve at the 100th cycle is much similar to that of the third one (Figure 2a, red line), which contains double reduction peaks at 1.34/1.2 V and a pair of oxidation peaks at 1.63/1.7 V, supporting the activation process with no change in the electrochemical reaction mechanism. The GCD curves before/after 100 cycles (Figure S3d,e, Supporting Information) also prove the enhanced Zn‐storage capability after the activation (the GCD curve after 100 cycles has a longer plateau than that of the 2nd/3rd cycle). The activation process is further investigated using the EIS plots acquired after the 2nd, 30th, and 100th cycles, as shown in Figure 2e. All the EIS plots are composed of a semicircle at the high/mid‐frequency region and a sloping line at the low‐frequency region. The diameter of the semicircle and the slope of the inclined line present the size of the charge‐transfer resistance and diffusion coefficient of Zn^2+^/H^+^ in the K‐MnO*_x_* electrode, respectively.^[^ ^43^ ^]^ The resistance after 2 cycles is 33.5 Ohms. Afterward, it changes to 40.1 Ohms after 30 cycles and 28.9 Ohms after 100 cycles. The slopes of the three lines are 0.60, 0.54, and 0.61 after 2, 30, and 100 cycles, respectively. This result suggests that the initial activation process delivers promoted diffusion coefficient of Zn^2+^/H^+^ and deceased resistance of charge transfer, which conjointly explain the improvement of electrochemical performance during the activation process.

The rate capability of K‐MnO*_x_* is displayed in Figure 2f. As shown, the K‐MnO*_x_* electrode delivers a discharge specific capacity of 303.1/242.1/183.8/147.8/125.4/116.4/107.6/100.7/94.3 mAh g^−1^ at 0.1/0.2/0.3/0.5/1.0/1.2/1.5/1.8/2.0 A g^−1^, respectively. Furthermore, the long‐cycling capacity of K‐MnO*_x_* (Figure 2g) remains at 60 mAh g^−1^ after 3000 cycles at the current density of 1.0 A g^−1^. The electrochemical performance, as shown in Figure 2h, is much better than that of the reported Mn‐based AZIBs cathode materials, such as MnO_2_,^[^ ^44^ ^]^ MnO_2_/MWCNT,^[^ ^45^ ^]^ layered MnO_2_,^[^ ^46^ ^]^ a‐MnO*_x_*,^[^ ^47^ ^]^ *δ*‐MnO_2_,^[^ ^48^ ^]^ *β*‐MnO_2_ nanorod,^[^ ^18^ ^]^ *δ*‐MnO_2_ NSs,^[^ ^37^ ^]^ and IO‐MnO_2_. ^[^ ^49^ ^]^ In addition, the electrochemical performances of K‐MnO*_x_* and K‐MnO_2_ are also compared. For the K‐MnO_2_ electrode, the shapes of CV/GCD curves in the initial three cycles (Figure S4a,b, Supporting Information) are very similar to those of K‐MnO*_x_* (Figure 2a,b), suggesting that the Zn‐storage mechanism for both of the materials is the same in the initial cycles. Nevertheless, the plateaus belonging to Zn‐storage (Figure S4c,d of the Supporting Information, between 1.35 and 1.0 V) quickly disappear with the increase of cycles and current densities, indicating a rapid decrease of Zn‐storage capability for K‐MnO_2_. Therefore, the cycling and rate performances of K‐MnO_2_ (Figure S4e,f, Supporting Information) are much weaker than that of K‐MnO*_x_* (Figure 2d,f). Moreover, after a comparison of the GCD curves of K‐MnO_2_ (Figure S4b, Supporting Information) and K‐MnO*_x_* (Figure 2b) electrodes in the initial three cycles, it is observed that the GCD‐curve shapes of K‐MnO_2_ and K‐MnO*_x_* are similar but minor different to some extent, especially at the end of the GCD discharge curve, which is attributed to the poorer Zn‐ion insertion/desorption reaction kinetics in K‐MnO_2_. This causes incomplete charge/discharge in the K‐MnO_2_ electrode. As a result, the shapes of the GCD curves are different, and also the performance of K‐MnO*_x_* is better than that of K‐MnO_2_.

To explain the reason for the weak electrochemical properties of K‐MnO_2_, EIS, GITT (galvanostatic intermittent titration technique), and CV data are collected, as shown in Figure S4g–i of the Supporting Information. First, the EIS plots verify that the K‐MnO_2_ electrode exhibits an increasing charge transfer resistance with the rising of cycles (Figure S4g, Supporting Information). Second, the GITT result (Figure S4h, Supporting Information) indicates that the K‐MnO_2_ electrode has a very low average Zn‐diffusion coefficient (log (D_Zn_) = −13 cm^2^ s^−1^).^[^ ^33^, ^50^ ^]^ Third, the peak current of CV curves (Figure S4i, Supporting Information) does not increase with the rising scan rate, indicating that no pseudocapacitance contributes to the specific capacity of K‐MnO_2_. Therefore, the large charge transfer resistance, the low Zn‐diffusion rate, and the lost pseudocapacitance contribution systematically result in the poor Zn‐storage properties of the K‐MnO_2_ electrode. In comparison with K‐MnO*_x_*, the only difference for K‐MnO_2_ is revealed in annealing, which results in the removal of crystal water and the phase transformation of K‐buserite into K‐contained MnO_2_ (JCPDS #44‐0141). Hence, it can be concluded that the crystal water and Zn‐buserite phase in K‐MnO*_x_* are crucial to the excellent Zn‐storage performance of K‐MnO*_x_*.

Furthermore, to understand the excellent performance of K‐MnO*_x_* in‐depth, the diffusion coefficient of Zn^2+^/H^+^ according to GITT is calculated (Figure 2i), and the pseudocapacitance contribution based on CV result is estimated (Figure 2j,k; Figure S3f, Supporting Information). The detailed principle for both calculations is reported in the Supporting Information (Equations (S1)–(S3)). The values of Log(D_Zn_) range from −10 to −12 during the discharge/charge process, and the average value is −10.6, which is relatively higher than that of the reported cases,^[^ ^33^, ^50^ ^]^ indicating a great Zn‐diffusion efficiency in the electrode. The pseudocapacitance contribution at the scan rate of 0.1/0.2/0.3/0.4/0.5 mV s^−1^ accounts for 69/74/79/84/89%. Therefore, it is inferred that the pseudocapacitance‐contribution percentage gradually increases with the rising scan rate, corresponding to the good rate capability of the K‐MnO*_x_* electrode. Summarily, the low charge transfer resistance, fast Zn‐diffusion efficiency, and high pseudocapacitance contribution collectively lead to the high Zn‐storage performance of K‐MnO*_x_*.

Inspired by the extraordinary pseudocapacitance contribution of K‐MnO*_x_* as an AZIBs cathode material, its electrochemical performance as the AZHSCs cathode material was further investigated. To assemble the AZHSCs device, AC was chosen as the anode material due to its excellent cycling stability as previously reported in the AC//Zn devices.^[^ ^15^, ^16^ ^]^ The AC sample was first investigated for its physicochemical characteristics and then for the electrochemical performance in AC//AC and AC//Zn devices, and thus it can match the K‐MnO*_x_* cathode with the AC anode better. The XRD pattern of AC (Figure S5a, Supporting Information) shows only a broad and weak (002) peak at 26° without any strong diffraction peak, indicating that the prepared AC is amorphous. According to the XPS survey spectrum (Figure S5b, Supporting Information), AC is composed of 4.2 at% O and 95.8 at% C without any peak that belongs to impurity. The fitted O 1s and C 1s spectra (Figure S5c,d, Supporting Information) reveal that O in AC primarily exists in C—O and C=O groups. Figure S5e of the Supporting Information is the Raman spectrum of AC, with strong G‐ and D‐band peaks. The intensity of the D‐ and G‐ bands (*I*_D_/*I*_G_) is 1.016, suggesting the very low content of crystalline graphite structure in AC, which well agrees with its XRD result.^[^ ^11^ ^]^ Based on the N_2_ adsorption–desorption analysis shown in Figure S5f of the Supporting Information, the average pore diameter of AC is evaluated to be 3.06 nm, and the pore volume (pores less than 187 nm at P/P^o^ = 0.99) reaches up to 0.41 cm^3^ g^−1^ with a 540.3 m^2^ g^−1^ *S*_BET_. In addition, SEM and TEM images (Figure S6a–e, Supporting Information) exhibit that AC is composed of many microspheres with a diameter of several hundred nanometers to micrometers. The TEM‐Mapping results (Figure S6f, Supporting Information) also reveal the low oxygen content in AC, and the SAED pattern (the inset in **Figure** **6**d) further confirms the amorphism of AC. In short, the AC microspheres with low oxygen content are amorphous with abundant inner pore structures and large specific surface area.

Symmetric AC//AC and asymmetric AC//Zn devices were investigated as follows to evaluate the electrochemical properties of AC. The CV curves of AC//AC at various scan rates from 2 to 250 mV s^−1^ (Figure S7a–c, Supporting Information) show a typical shape of electric double‐layer capacitors. The shape of these CV curves displays good symmetry, indicating the excellent stability of AC. However, the AC//AC device delivers relatively low specific capacitance (Figure S7d, Supporting Information) of 58.5/47.9/45.3 F g^−1^ at 0.1/1.0/2.0 A g^−1^. Besides, its power and energy density are also poor, as shown in Figure S7e,f of the Supporting Information. For instance, at 0.1/2.0 A g^−1^, its power density and energy densities are only 29.1/604.4 W kg^−1^ and 11.7/9.07 Wh kg^−1^, respectively. The weak specific capacitance, power density, and energy density fail to meet the increasing requirements for high‐performance energy‐storage devices. On the other hand, AC//Zn devices demonstrate better energy‐storage properties than AC//AC devices. Figure S8a,b of the Supporting Information is GCD curves of AC//Zn at various current densities from 0.1 to 3.0 A g^−1^. As seen, these curves exhibit good isosceles‐triangle shapes, indicating the good reversibility of AC. Hence, the AC//Zn displays good rate performance (Figure S8c–e, Supporting Information), delivering a specific capacitance of 219.6/195.2/182.6/171.1/164.9 /161.2/158.7/155.16 F g^−1^ at 0.1/0.3/0.5/1.0/1.5 /2.0/2.5/3.0 A g^−1^, with a power (energy) density of 41.9/127.5/212.3/424.8/635.6/850.2/1061.7/1419.1 W kg^−1^ (88.2/78.4/73.3/68.7/66.2/64.7/63.6/62.3 Wh kg^−1^). Although the data is better than that of AC//AC, it still cannot meet the need for both high energy density and high power density. But surprisingly, it is found that AC is quite stable in AC//Zn. As shown in Figure S8f of the Supporting Information, it delivers a specific capacitance of 161.2 F g^−1^ after 6000 cycles at 3.0 A g^−1^ with only a 7.1% decline during the whole process. Therefore, AC was selected as the anode material to assemble the K‐MnO*_x_*//AC devices.

**Figure 3:**
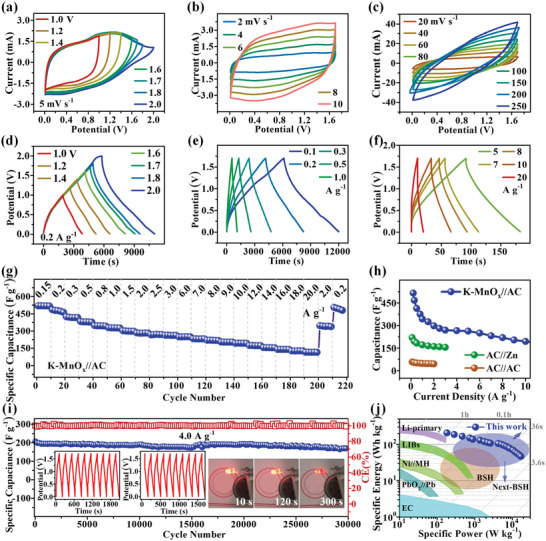
Electrochemical performance of K‐MnO*_x_* in K‐MnO*_x_*//AC. a) CV curves tested in diverse voltage windows. b,c) CV curves at various scan rates. d) GCD profiles in various voltage windows. e,f) GCD profiles at various scan rates. g) Rate performance at a different current density from 0.15 to 20 A g^−1^. h) Capacitance comparison of K‐MnO*_x_*//AC with AC//Zn and AC//AC at various current densities. i) Long‐cycling performance of K‐MnO*_x_*//AC at 5.0 A g^−1^. The inset GCD curves are from the initial ten cycles (left) and the ten cycles after 20 000 cycles (right), and the inset photo pictures show that a K‐MnO*_x_*//AC device lights up an LED bulb for more than 5 min. j) Ragone plots of K‐MnO*_x_*//AC, and comparison with other electrochemical energy storage systems, including electrochemical capacitors (EC), primary lithium batteries (Li‐primary), lithium‐ion batteries (LIBs), nickel–metal hydride batteries (Ni//MH), PbO_2_–Pb batteries (PbO_2_//Pb), battery‐supercapacitor hybrid (BSH) devices, and next‐generation battery‐supercapacitor hybrid (Next‐BSH) devices.

The electrochemical properties of K‐MnO*_x_*//AC (**Figure** **3**a–j, Supporting Information) were further investigated. As is known, the voltage window is an important parameter for the performance of K‐MnO*_x_*//AC devices, which can significantly affect the electrochemical performance. A suitable voltage window is chosen according to the CV and GCD curves collected in the voltage range from 1.0 to 2.0 V. As seen in Figure 3a–d, the area of CV and GCD curves increases gradually with the rising of the voltage window, whereas their symmetry begins to change when the voltage range surpasses 1.7 V. Therefore, the voltage window of 0.01–1.7 V is set as the voltage window for the test of the K‐MnO*_x_*//AC device.

Figure S9a of the Supporting Information is a representative CV curve of K‐MnO*_x_*//AC at 2.0 mV s^−1^. The CV curve displays a good rectangular shape without any apparent peaks ascribed to the battery behavior, suggesting that the capacitive behavior dominates in K‐MnO*_x_*//AC. The CV curves at various scan rates (1–250 mV s^−1^) are shown in Figure 3b,c, and Figure S9a (Supporting Information). The rectangular CV shape can be well kept even at 10 mV s^−1^, indicating good stability and excellent rate performance of K‐MnO*_x_*//AC. When the scan rate increases to 250 from 20 mV s^−1^, the CV shape changes gradually to a nonideal rectangular, which is associated with the asymmetric structure of K‐MnO*_x_*//AC. More specifically, the asymmetric energy‐storage mechanism is revealed on the AC anode and K‐MnO*_x_* cathode. In addition, the CV shape does not deform severely even at the highest scan rate of 250 mV s^−1^, indicating that both of the electrode materials exhibit fast energy‐storage dynamics during cycling. Moreover, GCD curves of K‐MnO*_x_*//AC acquired at various current densities between 0.1 and 20 A g^−1^ (Figure 3d,e) keep good equilateral triangle shapes, which further indicates good stability and reversibility.

The specific capacitance of K‐MnO*_x_* was evaluated by GCD testing at various current densities (Figure 3g), and the values of specific capacitance (calculated using Equation (1)) are 515.0, 467.3, 413.7, 343.2, 297.8, 269.1, 262.7, 219.2, 194.1, 173.3, 155.4, 140.6, 127.6, and 116.0 F g^−1^ at 0.15, 0.3, 0.5, 1.0, 2.0, 3.0, 5.0, 8.0, 10.0, 12.0, 14.0, 16.0, 18.0, and 20.0 A g^−1^, respectively. This performance of K‐MnO*_x_*//AC is superior to that of AC in AC//Zn and AC//AC devices (Figure 3h), as well as some reported materials (Table S2, Supporting Information). In addition, K‐MnO*_x_* also exhibits excellent cycling stability, as shown in Figure 3i. It can maintain 92.9%/81.8% after 20 000/30 000 cycles at 5.0 A g^−1^ with near 100% CE during all the cycles. The inset of GCD curves in Figure 3i is from the initial ten cycles (left) and the 10 000–10 010 cycles (right). The shape of these curves is very similar, indicating good stability. Besides, the photo pictures (the inset in Figure 3i) show that a K‐MnO*_x_*//AC device can light up an LED bulb for more than 5 min, demonstrating a promising application potential. Furthermore, the power density (*P*) and energy density (*E*) of K‐MnO*_x_*//AC were calculated. The highest power/energy density reaches 16.94 kW kg^−1^ (at 20 A g^−1^)/206.7 Wh kg^−1^ (at 0.15 A g^−1^), respectively. The power density and energy density are much superior to that of AC//Zn, AC//AC (Figure S9c, Supporting Information), and some previously reported devices (Table S2, Supporting Information), which can meet the increasing requirement for the next‐generation hybrid capacitors, as shown in Ragone plots (Figure 3j).^[^ ^2^, ^15^ ^]^

The electrochemical energy‐storage mechanism of K‐MnO*_x_* is investigated via a series of ex situ analyses (XPS, Raman, XRD, SEM, and TEM–EDS–SAED). Before these analyses, the data of K‐MnO*_x_* electrodes in various discharge–charge states were obtained, including pristine, first discharging to 1.2 V (1st‐1.2 V)/1.0 V (1st‐1.0 V), and first charging to 1.6 V (1st‐1.6 V)/1.8 V (1st‐1.8 V), as well as full‐charging (100th‐1.0 V)/discharging (100th‐1.8 V) in the 100th cycle. To eliminate the side effects of electrolyte salt, K‐MnO*_x_* electrodes in all these states were washed by deionized water (DIW) before testing.

**Figure 4:**
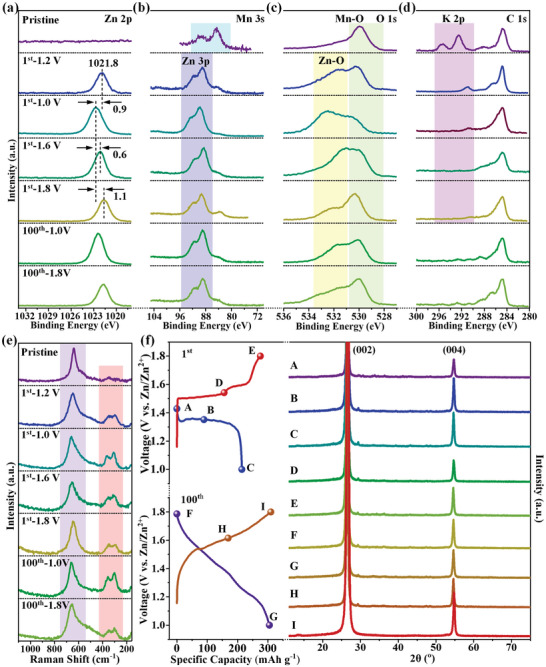
a–e) Ex situ XPS and Raman spectra of K‐MnO*_x_* electrodes in various states, including pristine, first discharging to 1.2 V (1st‐1.2 V)/1.0 V (1st‐1.0 V), first charging to 1.6 V (1st‐1.6 V)/1.8 V (1st‐1.8 V), and full‐charge (100th‐1.0 V)/discharge (100th‐1.8 V) in the 101st cycle. f) The first/hundredth GCD curves, and corresponding XRD patterns of K‐MnO*_x_* electrodes in the marked states (A–I).

**Figure** **4a–e** and Figure S10a–d (Supporting Information) show ex situ XPS and Raman spectra of K‐MnO*_x_* electrodes in various charge–discharge states. As seen in Figure S10a,b of the Supporting Information, no signal of S 2p is detected in all these spectra, suggesting the full removal of electrolyte salt (Zn(CF_3_SO_3_)_2_ and Mn(CF_3_SO_3_)_2_) upon the washing process. Strong Mn 2p and Zn 2p peaks (Figure S10c,d, Supporting Information) are visible in diverse charge/discharge states, indicating the electrochemical reaction products of K‐MnO*_x_* are insolvable in DIW. During the first discharge to 1.2 and 1.0 V (Figure 4a; Figure S10a, Supporting Information), a Zn 2p_3/2_ peak appears at 1021.8 eV (1st–1.2 V), followed by a gradual increase of peak intensity and a shift of peak position to 1022.7 eV (1st–1.0 V), indicating the insertion of Zn^2+^ into K‐MnO*_x_* during this process. After the Zn^2+^ insertion, the Mn 3s peaks of K‐MnO*_x_* (Figure 4b) are covered by Zn 3p peaks, and the O 1s peak of K‐MnO*_x_* (O‐Mn) (Figure 4c) is covered by the O 1s peak of O–Zn. In addition, K 2p peaks (Figure 4d) disappear with the insertion of Zn^2+^. The reason for this can be summarized as follows. The bivalent Zn^2+^ with strong electrostatic interaction occupies the position of K in K‐buserite. Thus, the K atom is free and can be washed out by DIW. All these results verify that K‐MnO*_x_* (which is composed of K‐buserite units) is transformed into Zn‐buserite in the first discharge process. During the first charge cycle, the Zn 2p_3/2_ peak shifts to the lower binding energy of 1021.6 eV (Figure 4a, 1st‐1.8 V), and the peak intensity of the Zn 3p/O 1s (O‐Zn) peaks decreases (Figure 4b,c, 1st‐1.6 V and 1st‐1.8 V), which are attributed to the Zn^2+^ extraction from Zn‐buserite. However, in the first charge state (1st‐1.8 V), the peaks of Zn 2p and Zn 3p do not disappear, suggesting that the transformation of Zn‐buserite is irreversible.

Raman spectra in all these states display a strong peak between 630 and 760 cm^−1^, which is attributed to the characteristic Mn–O stretching vibration of MnO_6_ units. The peak does not disappear in full‐discharge/charge states, indicating that MnO_6_ units in K‐MnO*_x_* are not destroyed in the phase‐transformation process of K‐buserite into Zn‐buserite. The peaks between 250 and 400 cm^−1^ in Raman spectra are attributed to the Zn–O vibration in Zn‐buserite.^[^ ^15^, ^35^ ^]^ During the first discharge, the intensity of Zn–O peaks increases due to the gradual insertion of Zn^2+^. In the following charge process, the peaks do not disappear fully because of the irreversible transformation of Zn‐buserite, agreeing well with the above XPS results. After 100 discharge–charge cycles, all the XPS and Raman spectra keep a similar trend to the corresponding state in the first cycle, indicating the high stability of Zn‐buserite. Furthermore, the crystalline information of Zn‐buserite was investigated using ex situ XRD, as shown in Figure 4f. Both of the strong diffraction peaks at 26° and 55° are attributed to the (002) and (004) planes of graphite structure in CB and graphite foil (for ex situ XRD testing, K‐MnO*_x_* was coated on graphite foil). During the first discharge/charge, no peak that belongs to Zn‐buserite can be observed, suggesting that the transformed Zn‐buserite is also amorphous, like K‐MnO*_x_* (K‐buserite). Even going through 100 cycles, the Zn‐buserite still keeps the amorphous character, indicating high stability.

**Figure 5:**
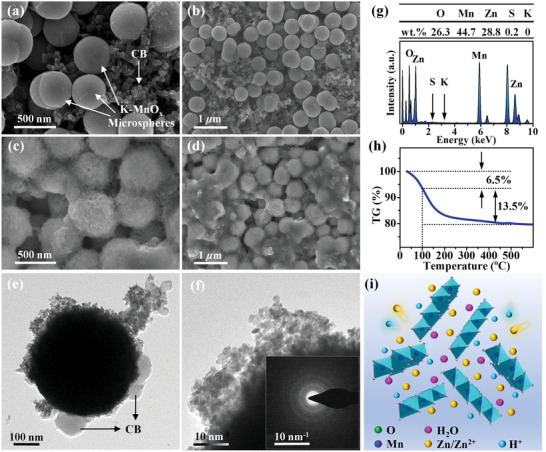
a,b) SEM images of pristine K‐MnO*_x_* electrode. c,d) SEM images, e,f) TEM‐SAED images, g) TEM‐EDS spectrum, and h) TGA curves of K‐MnO*_x_* electrode material after 100 GCD cycles at 0.2 A g^−1^. i) Schematical illustration for the Zn‐storage mechanism of transformed amorphous Zn‐buserite in K‐MnO*_x_*//Zn.

The morphology and structure of the K‐MnO*_x_* electrode were further investigated using SEM and TEM–EDS–SAED. In the SEM images of the pristine K‐MnO*_x_* electrode (**Figure** **5**a,b), K‐MnO*_x_* microspheres and CB (carbon black) particles can be observed. After 100 GCD cycles in K‐MnO*_x_*//Zn, the microspherical structure is still visible (Figure 5c,d), but these microspheres are already Zn‐buserite instead of K‐MnO*_x_* (K‐buserite), due to the K‐buserite has been transformed into Zn‐buserite in the first discharge according to above XPS–Raman–XRD analyses. The good shape of Zn‐buserite can be maintained after 100 cycles, indicating the good stability of the K‐MnO*_x_* electrode. The surface of these Zn‐buserite microspheres is shown in Figure 5c, and it is rather different from that of K‐MnO*_x_*:Zn‐buserite microspheres present a rougher surface with many visible particles. These particles are irregular and in nanoscale, which can be confirmed by TEM images (Figure 5e,f; Figure S11a–c, Supporting Information). Hence, it is concluded that Zn‐buserite microspheres are composed of a large number of irregular nanoparticles. Moreover, the SAED pattern (the inset in Figure 5f) obtained from a Zn‐buserite microsphere does not display any diffraction spot/cycle, suggesting the amorphous state of Zn‐buserite. Due to the existence of short‐range‐order structures in amorphous K‐MnO*_x_* according to the above XRD analysis (Figure 1a), it can be inferred that short‐range‐order structure also exists in amorphous Zn‐buserite, and this short‐range‐order structure is too small/irregular to lead to any clear XRD diffraction peak and SAED pattern. However, these short‐range‐order structures in amorphous Zn‐buserite still have the same crystalline interplanar distance (≈0.68 nm) as the reported crystalline Zn‐buserite.^[^ ^15^, ^32^ ^]^ The large interplanar distance is more than fivefold of the Zn^2+^ diameter (0.134 nm), benefiting the diffusion of Zn^2+^. Furthermore, the EDS spectrum (Figure 5g) acquired from a single Zn‐buserite microsphere exhibits strong signals of elemental Mn, O, and Zn. According to the EDS result, weight percentage values of O, Mn, Zn, S, and K elements are calculated to be 26.3, 44.7, 28.8, 0.2, and 0, verifying that the Zn‐buserite phase does not contain the K element. Therefore, K‐MnO*_x_* (K‐buserite) is fully transformed into Zn‐buserite. The Zn‐buserite with numerous short‐range‐order structures has many advantages in Zn‐storage. First, it possesses abundant structural defects that can provide more active sites for Zn‐storage, leading to a high specific capacity. Second, it has the character of intrinsic isotropic nature, which effectively relieves the stress caused by the insertion of Zn^2+^, resulting in superior cycling stability. Finally, the amorphous Zn‐buserite, different from its crystalline counterparts, can furthest shorten the Zn^2+^ diffusion path due to their minuscule atomic arrangement units, enabling the highly efficient diffusion of Zn^2+^.

To investigate the valence state variation of manganese after activation, we further analyzed the Mn 3s and Mn 2p XPS spectra of the K‐MnO*_x_* electrode at the full‐charge (100th‐1.8 V) and discharge (100th‐1.0 V) state after 100 cycles (Figure S11d–i, Supporting Information). According to the Mn 3s/Mn 2p spectra (Figure S11d,e, Supporting Information) and the fitting result of Mn 2p (Figure S11f,g, Supporting Information), the change of valence state of manganese elements is small with the coexistence of Mn^3+^ and Mn^4+^ in Zn‐buserite, no matter in full discharge or full charge state. The splitting energy of Mn 3s is 4.3 and 4.2 eV in fully discharged and charged K‐MnO*_x_* electrodes, respectively, as shown in Figure S11h,i of the Supporting Information. Compared with the splitting‐energy value of the original K‐MnO*_x_* material (4.9 eV, in Figure 1f), the splitting energy of Mn 3s after 100 cycles decreases significantly, indicating an increased Mn oxidation state after the charge of K‐MnO*_x_* phase to Zn‐MnO*_x_* phase.^[^ ^32^ ^]^ However, according to the fitted Mn 3s result with a slight variation of splitting energy, the change of Mn oxidation state in K‐MnO*_x_* after 100 cycles is minor during the discharge/charge process.

In addition, the content of crystal water in the transformed Zn‐buserite was tested by TGA, as shown in Figure 5h. The weight percentage of crystal water is calculated to be ≈13.5%, which is lower than that in K‐MnO*_x_*. The loss of crystal water may happen in the phase transformation process, and it can meanwhile provide more active sites for Zn‐storage. As reported, crystal water in Zn‐buserite is beneficial to Zn‐storage by eliminating the strong electrostatic interaction of bivalent Zn^2+^ with host materials.^[^ ^34^, ^51^ ^]^ According to all the ex situ analysis results, Zn‐buserite and its Zn‐storage mechanism in K‐MnO*_x_*//Zn devices can be schematically illustrated in Figure 5i. In brief, the Zn‐buserite with high activity and stability is irreversibly transformed from K‐buserite (K‐MnO*_x_*) in the first discharge process, which then participates in the following electrochemical reaction, enabling the high‐performance Zn‐storage of K‐MnO*_x_* electrode.

To investigate the volume expansion in Mn‐based cathode materials, the volume trend of the K‐MnO*_x_* after 100 cycles is analyzed as follows. Based on our test, the atomic ratio of Zn to Mn in the full‐charged Zn‐buserite is 0.541: 1 (transformed from the weight percentage in Figure 5g), while the atomic ratio of K to Mn in the K‐buserite is 0.186:1 (Table S1, Supporting Information). The former ratio is much higher than the latter one. Therefore, the volume of Zn‐buserite, in theory, is larger than that of K‐buserite. In addition, it is also observed that the diameter of Zn‐buserite is bigger than that of K‐buserite according to SEM images in Figure 5a,c. Due to the diameter of K‐buserite being relatively uniform, as shown in Figure 1e,f, the conclusion is reliable. Hence, it is concluded that the volume of the K‐MnO*_x_* electrode increases after 100 cycles. On the other hand, the volume of Zn‐buserite will increase during a discharge process with the insertion of Zn^2+^ and H^+^ into the host material, while the decrease of the host‐material volume will occur during a charging process after Zn^2+^/H^+^ extraction. Therefore, the volume of the active material in the full discharge state is larger than that in the full charge state.

The Zn‐buserite with high activity/stability benefits a high‐efficient Zn‐storage, but the growth of Zn‐dendrites on Zn anode is a main issue for the K‐MnO*_x_*//Zn devices, which can result in short‐circuit and poor cycling stability. The suppression of Zn‐dendrites in AZIBs is still a challenge at present, and so does in the AC//Zn devices. The severe Zn‐dendrites are revealed by a comparison observation of the Zn anode, as shown in Figure S12a–i of the Supporting Information. For the Zn anodes matched with K‐MnO*_x_* and AC, clear Zn‐dendrites can be observed after 100 cycles at 0.2 A g^−1^, indicating that the growth of Zn‐dendrites is unavoidable in the two systems (K‐MnO*_x_*//Zn and AC//Zn). However, this issue can be effectively addressed in the K‐MnO*_x_*//AC system, which will be further discussed in the following.

**Figure 6:**
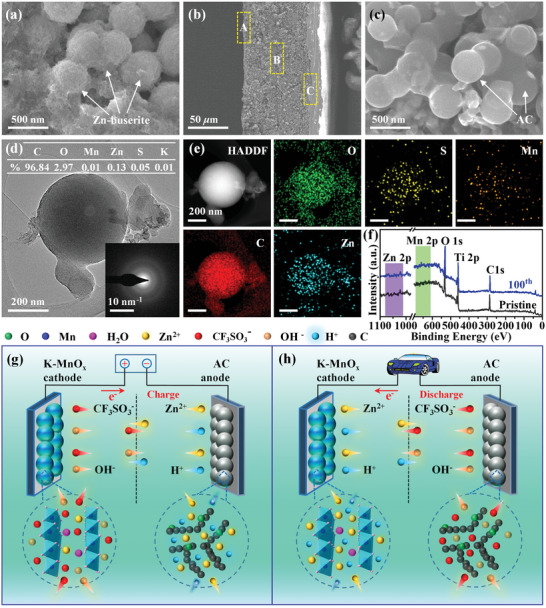
a) SEM image of K‐MnO*_x_* electrode in K‐MnO*_x_*//AC. b) SEM image (cross‐section) of a full‐charge‐state AC electrode in K‐MnO*_x_*//AC, where the three representative areas marked A, B, and C will be investigated further to deeply understand the energy‐storage mechanism of K‐MnO*_x_*//AC. c) SEM image of the surface of AC electrode (area A). d) TEM‐SAED and e) mapping images of AC electrode (area B). f) XPS survey spectra acquired from the surface of the Ti collector (marked in area C, blue spectrum) and pristine foil (black). Schematical illustration of the energy storage mechanism of K‐MnO*_x_*//AC devices in g) charge and h) discharge processes.

**Figure** **6**a and Figure S13a,b (Supporting Information) show the surface morphology of K‐MnO*_x_* cathode in K‐MnO*_x_*//AC after 100 cycles at 2.0 A g^−1^. As seen, the K‐MnO*_x_* electrode keeps the same morphology as that in K‐MnO*_x_*//Zn (Figure 5c,d), verifying the good stability of K‐MnO*_x_* in K‐MnO*_x_*//AC. At the same time, the AC anode was investigated. Figure 6b is the SEM image of the AC electrode (cross‐section) with three areas marked A (the surface of the AC electrode), B (inner AC electrode), and C (the surface of the Ti collector), which are further investigated by SEM, TEM‐SAED‐EDS‐Mapping, and XPS. As shown in Figure 6c and Figure S13c,d (Supporting Information), the AC electrode in K‐MnO*_x_*//AC does not change visibly, and no Zn‐dendrite is observable in comparison with the pristine AC electrode (Figure S14a–c, Supporting Information). Hence, combining the SEM images of AC and K‐MnO*_x_* electrodes, it is concluded that both of the electrodes in K‐MnO*_x_*//AC are very stable and well‐matched. More importantly, the Zn‐dendrites issue in K‐MnO*_x_*//AC is effectively eliminated. This is a major reason for the superior cycling stability of the device.

To explain the reason for no change of AC morphology after 100 cycles, the surface of the AC electrode matched with the Zn anode is observed by SEM, as shown in Figure S14d–f of the Supporting Information. The surface of the AC cathode in AC//Zn does not change significantly after 100 cycles at 0.2 A g^−1^, indicating the good stability of AC. Unlike the previous reports^[^ ^12^, ^52^ ^]^ with sulphate (ZnSO_4_ and MnSO_4_) as an electrolyte, no crystalline structure of Zn_4_SO_4_(OH)_6_ 5H_2_O or ZnSO_4_·3Zn(OH)_2_·5H_2_O on the surface of AC electrode was found, because the electrolyte (2 m Zn(CF_3_SO_3_)_2_ + 0.2 m Mn Zn(CF_3_SO_3_)_2_) chosen in this work does not include SO_4_^2−^. Therefore, in the selected electrolyte system and operating voltage range, these crystalline products did not produce during the repeated GCD test. This is a crucial factor that leads to superior cycling stability.

Moreover, AC anode matched with K‐MnO*_x_* was further studied by TEM‐EDS‐Mapping in Figure 6d,e. Before TEM observation, a charging to 1.8 V (0.2 A g^−1^) on the AC anode was carried out. During the charging process, all positive ions (Zn^2+^, Mn^2+^, and H^+^) were pushed into the AC anode, and negative ions (OH^−^ and (CF_3_SO_3_)_2_^2−^) were forced into the K‐MnO*_x_* cathode. After the charging process, the AC anode was dispersed into *N*‐methyl pyrrolidone (NMP) solution to dissolve the polyvinylidene difluoride (PVDF) binder and then transferred onto a TEM grid before observation. The TEM‐EDS‐Mapping images demonstrate strong signals of elemental C and O, as well as very weak signals of Zn, Mn, and S. The atomic proportion of Zn/Mn/S is calculated to be 0.13/0.01/0.05, according to the EDS‐Mapping results in Figure 6d. The very low ratio of Zn, Mn, and S is due to that most of the electrolyte was washed by NMP, which supports the adsorption mechanism of AC, namely, positive/negative ions were adsorbed on AC during the charge/discharge. The adsorption process plays a crucial role in the energy‐storage process. Besides, the content of Zn is much higher than that of Mn and S, because of the strong attraction force of the O atom in AC to Zn^2+^. Specifically, some O atoms in AC can combine Zn^2+^ and then form a strong O—Zn bond, which is difficult to dissolve in NMP.

As reported in the AC//Zn and K‐MnO*_x_*//Zn systems (Figure S12, Supporting Information), Zn ions were deposited on the Zn anode in the charging process, and hence severe Zn‐dendrites occur. In the K‐MnO*_x_*//AC system, to verify whether the Zn‐dendrites grow on the Ti collector of the AC anode, XPS testing was conducted. Before the test, the AC anode coated on Ti foil was washed off with NMP. As shown in Figure 6f, the XPS survey spectra of pristine Ti foil (black) and Ti collector of AC anode (blue, going through 100 GCD cycles) show no clear Zn 2p (1010–1060 eV) and Mn 2p (630–660 eV) peak, verifying no Zn‐dendrites on Ti collector. Therefore, it is concluded that these positive ions like Zn^2+^ were fully adsorbed onto AC during the charging process, and then desorbed out of AC during the discharge process. Accordingly, combining the Zn‐storage of K‐MnO*_x_* in K‐MnO*_x_*//Zn, the energy‐storage mechanism of a K‐MnO*_x_*//AC device is schematically illustrated in Figure 6g,h. Briefly, all the positive ions are forced toward and adsorbed onto the AC anode during the charging process, and these positive ions reverse into the K‐MnO*_x_* cathode during the discharge process. The migration direction of negative ions is inverse to the positive ions during the whole GCD cycle. On the other hand, when the Zn^2+^ and H^+^ are driven into the K‐MnO*_x_* cathode, some of them are inserted into the inner structure of Zn‐busesrite, and others are adsorbed onto Zn‐busesrite frames, enabling high‐performance Zn‐storage of K‐MnO*_x_* electrode in K‐MnO*_x_*//AC. In short, the adsorption mechanism plays a crucial role in the energy storage of K‐MnO*_x_*//AC devices.

## 3 Conclusions

In summary, we synthesized an amorphous K‐buserite microspheres (K‐MnO*_x_*) cathode material for both of AZIBs and AZHSCs. The energy‐storage mechanism was revealed via a series of ex situ analyses. It suggests that the amorphous K‐buserite phase is irreversibly transformed into amorphous Zn‐buserite in the first discharge process, which subsequently participates in the following electrochemical reactions. The transformed Zn‐buserite phase possesses the advantages of amorphous MnO*_x_* and crystalline K‐buserite, and exhibits superior energy‐storage performance, including high rate capability and long cycling lifespan. Particularly, the K‐MnO*_x_* electrode delivers a specific capacitance of 116 F g^−1^ at 20.0 A g^−1^ with the discharge/charge process being finished within 15 min. The power and energy density of K‐MnO*_x_*//AC devices can reach as high as 16.94 kW kg^−1^ (at 20 A g^−1^) and 206.7 Wh kg^−1^ (at 0.2 A g^−1^), respectively. In addition, the K‐MnO*_x_* electrode in K‐MnO*_x_*//AC maintains 92.9% capacitance retention after 20000 cycles at 5.0 A g^−1^. Therefore, the K‐MnO*_x_*//AC device that incorporates the virtues of AZIBs and ASCs (aqueous supercapacitors) can meet the requirements for both high energy and power density, which might provide a new choice for the design of high‐performance energy‐storage equipment.

## 4 Experimental Section

### Materials

Potassium permanganate (KMnO_4_, 99.99%), concentrated ammonia solution (NH_3_·H_2_O, ≈30.0%), glucose monohydrate (C_6_H_12_O_6_·H_2_O), and NMP were purchased from the Shanghai Aladdin Bio‐Chem Technology Co., LTD. Zinc (II) trifluoromethanesulfonate (Zn(CF_3_SO_3_)_2_, >99.8%), and Manganese (II) trifluoromethanesulfonate (Mn(CF_3_SO_3_)_2_, > 98%) were brought from the Bide Pharmatech Co., LTD. All these raw chemicals were used without further purification. Titanium foil, zinc foil, Whatman GF/D separator, PVDF, and stainless‐steel cell‐assembling gadgets (battery cases, springs, and shims) were acquired from the Kelude Experimental Equipment Technology Co., LTD. DIW was made by a laboratory ultrapure water system made by the Laje Intelligent Precision Instruments Co. LTD.

### Preparation of K‐MnO*_x_* and K‐MnO_2_ Microspheres

The K‐MnO*_x_* was synthesized through a coprecipitation method with an ammonia‐reduction process.^[^ ^36^ ^]^ Specifically, ammonia solution (180 g, ≈28.0%) and KMnO_4_ powder (0.5 g) were added into a polytetrafluoroethylene (PTFE) reactor with a sealing cover to form a mixture, and then the mixture was sealed and intensely stirred for 15 min to dissolve KMnO_4_ into an ammonia solution. Subsequently, the mixed solution was stood in darkness for 24 h without any stirring. Finally, the brown K‐MnO*_x_* was collected after filtering with a 0.22 µm PTFE filter, washing with DIW, and drying at 60 °C overnight. Afterward, half of the K‐MnO*_x_* sample was transformed into K‐contained MnO_2_ (named K‐MnO_2_) via annealing at 500 °C for 2 h in an Ar atmosphere. During this annealing process, crystal water in K‐MnO*_x_* was removed, and K‐MnO*_x_* was transformed into K‐MnO_2_.

### Preparation of Activated Carbon Microspheres

Activated carbon (AC) was synthesized using a simple hydrothermal method with glucose as a carbon source, followed by a KOH‐activation process. Specifically, a glucose solution (5.4 g glucose and 60 g DIW) was heated at 190 °C for 8 h in a 100 mL PTFE hydrothermal reactor, and then some carbon precursor was collected after filtering, drying at 90 °C for 24 h, and annealing at 550 °C for 5 h in high‐pure N_2_. Afterward, the carbon precursor was activated using KOH at 700 °C for 3 h in an Ar atmosphere. The mass ratio of carbon precursor to KOH is 1:2. Finally, the AC microspheres were acquired after washing out KOH with 1 m HCl aqueous solution, and drying at 100 °C for 24 h.

### Materials Characterization

Crystalline information was evaluated by XRD using a Bruker D8 Advance diffractometer (Cu K*α*, *λ* = 1.54 Å, 5° min^−1^). Structural and compositional information were investigated by Raman microscopy (Thermo DXR3xi with a 532 nm light source) and XPS in a PHI‐5000 VersaProbe III system (Al K*α*, mono 1486.6 eV, pass energy of 69/280 eV for elements/survey spectra). The morphology and structure of samples were revealed by a Regulus 8100 scanning electron microscopy (accelerating voltage 20 kV), an FEI Tecnai G2 Spirit TWIN transmission electron microscopy (200 kV), and an FEI‐Talos F200X high‐resolution transmission electron microscopy (HRTEM, 200 kV). SAED patterns and EDS spectra were tested in the FEI Tecnai G2 Spirit TWIN TEM. HAADF and annular bright field images were acquired in the FEI‐Talos F200X HRTEM system under the STEM mode. Brunauer–Emmett–Teller specific surface area (*S*_BET_) and pores distribution were analyzed by nitrogen adsorption–desorption testing on a V‐Sorb 2800P analyzer. Water content in samples was evaluated via TGA in the N_2_ atmosphere using a PerkinElmer TGA 8000 system.

### Electrochemical Measurements

The electrochemical performance was tested in 2032 coin cells. First, electrodes were prepared by coating active materials (including K‐MnO*_x_*, K‐MnO_2_, and AC) on Ti plates using PVDF as a binder and NMP as a solvent. Taking preparation of K‐MnO*_x_* electrodes as an example, K‐MnO*_x_* (80 wt%), conductive carbon black (CB, 10 wt%), and PVDF binder (10 wt%) were mixed in an agate mortar to form a uniform paste, and then the paste was coated on Ti plates. After drying at 60 °C overnight, the prepared K‐MnO*_x_* electrodes with a mass loading of more than 1.5 mg cm^−2^ were collected. Similarly, the K‐MnO_2_ and AC electrodes were made in the same way. Afterward, a coin cell was assembled in the ambient atmosphere with 2 m Zn(CF_3_SO_3_)_2_ + 0.2 m Mn(CF_3_SO_3_)_2_ as an electrolyte, and GF/D glass microfiber filter as a separator. A piece of pure Zn foil was used as an anode for assembling K‐MnO*_x_*//Zn and AC//Zn devices. For K‐MnO*_x_*//AC devices, the K‐MnO*_x_* and AC were cathode and anode, respectively. Before assembling K‐MnO*_x_*//AC devices, both K‐MnO*_x_* and AC electrodes were preactivated in coin cells using Zn foil as anode for 100 cycles at 0.2 A g^−1^. To keep the balance of batteries, the mass ratio of K‐MnO*_x_* to AC was 1: 3.

GCD tests were conducted on Land (CT2001A) and Neware (CT‐4008T) battery testing systems to obtain data on cycling performance, rate capability, and GITT. CV curves and electrochemical impedance spectroscopy (EIS) plots were acquired on a Wuhan CorrTest electrochemical workstation. The frequency range for EIS testing is 10^5^–10^−2^ Hz. All the tests were conducted at room temperature. The specific capacitance (*C*), power density (*P*), and energy density (*E*) of supercapacitors were calculated using Equations (1)–(3)

(1) $$C=Itm^{-1}\;\Delta U^{-1}$$

(2) $$P=I\;\Delta U{\left(4m\right)}^{-1}$$

(3) $$E=It\Delta U{\left(4m\right)}^{-1}$$

where ^1)^ *C*, ^2)^ *I*, ^3)^ *t*, ^4)^ *m*, ^5)^Δ*U*, ^6)^ *P*, and ^7)^ *E* represent ^1)^specific capacitance (F g^−1^), ^2)^GCD current (A), ^3)^discharging time (s), ^4)^the loading mass of electrode materials (g), ^5)^potential window (V), ^6)^power density (W kg^−1^), and ^7)^energy density (Wh kg^−1^), respectively.

## Conflict of Interest

The authors declare no conflict of interest.

## Supporting information

Supporting Information

## Data Availability

The data that support the findings of this study are available from the corresponding author upon reasonable request.
