# Supplementary material for: Amorphous K‐Buserite Microspheres for High‐Performance Aqueous Zn‐Ion Batteries and Hybrid Supercapacitors
Source: Adv Sci (Weinh). 2023 Feb 24;10(13):2207329. doi: 10.1002/advs.202207329 (PMC10161118; doi:10.1002/advs.202207329)
Supplement: Supplementary file 1 — Supporting Information [file ADVS-10-2207329-s001.pdf]

## Supporting Information (SI) for:

# Amorphous K-buserite microspheres for high-performance aqueous Zn-ion batteries and hybrid supercapacitors

*Zhi-Qiang Wang*[a,b,*], *Hong-Ming Chen*[a,b], *Xiao-Dong Liu*[a,b], *Li-Ying Song*[a,b], *Bu-Sheng Zhang*[a,b], *Yun-Guo Yang*[a], *Zhao-Cheng Zhang*[c], *Qian Li*[d], *Tian-Qi Gao*[c], *Jing Bai*[a,b], *Woon-Ming Lau*[a,b,*], *and Dan Zhou*[a,b,*]

[a] Beijing Advanced Innovation Center for Materials Genome Engineering & Center for Green Innovation, School of Mathematics and Physics, University of Science and Technology Beijing, Beijing 100083, China

[b] Shunde Innovation School, University of Science and Technology Beijing, Foshan, Guangdong 528000, China

[c] Center for Electron Microscopy and Tianjin Key Laboratory of Advanced Functional Porous Materials, Institute for New Energy Materials & Low-Carbon Technologies, School of Materials Science and Engineering, Tianjin University of Technology, Tianjin 300384, China

[d] The Center of New Energy Materials and Technology, School of Materials Science and Engineering, Southwest Petroleum University, Chengdu, Sichuan 610500, China

*Corresponding authors:

b20200368@xs.ustb.edu.cn (Z. Q. Wang), leolau@ustb.edu.cn (W. M. Lau) and zhoudan@ustb.edu.cn (D. Zhou)

## The principle of pseudocapacitance contribution calculation

The pseudocapacitance contribution ratio can be evaluated by the following formulas:[1, 2]

$$\log i = b \log v + \log a \ (i = av^{b}) \quad \text{(Eq. S1)}$$

$$i(V)/v^{0.5} = k_1 v^{0.5} + k_2 \ (i(V) = k_1 v + k_2 v^{0.5}) \quad \text{(Eq. S2)}$$

Where, *v* and *i* are the scan rate and peak current, respectively. Besides, *a*, *b*, $k_1$ and $k_2$ are adjustable parameters when linear fitting.

## The principle of Zn-ion diffusion coefficient calculation

The Zn-ion diffusion coefficient can be calculated using GITT data based on Fick's second law.[3, 4]

Fick's second law is described by the following equation.

$$D_{Zn^{2+}} = \frac{4}{\pi\tau}\left(\frac{m_B V_M}{M_B S}\right)^2\left(\frac{\Delta E_S}{\Delta E_t}\right)^2 \quad \text{(Eq. S3)}$$

Where, $^{1)}\tau$, $^{2)}m_B$, $^{3)}V_M$, $^{4)}M_B$, $^{5)}S$, $^{6)}\Delta Es$, and $^{7)}\Delta Et$ represent $^{1)}$the constant current pulse time, $^{2)}$the weight of the active material, $^{3)}$the molar volume of the active material, $^{4)}$the molar mass of the active material, $^{5)}$the active surface area of the electrolyte-electrode interface, $^{6)}$the voltage change between the steady and original states at the plateau potential, and $^{7)}$the total voltage-change during the current pulse time τ excluding the iR drop.

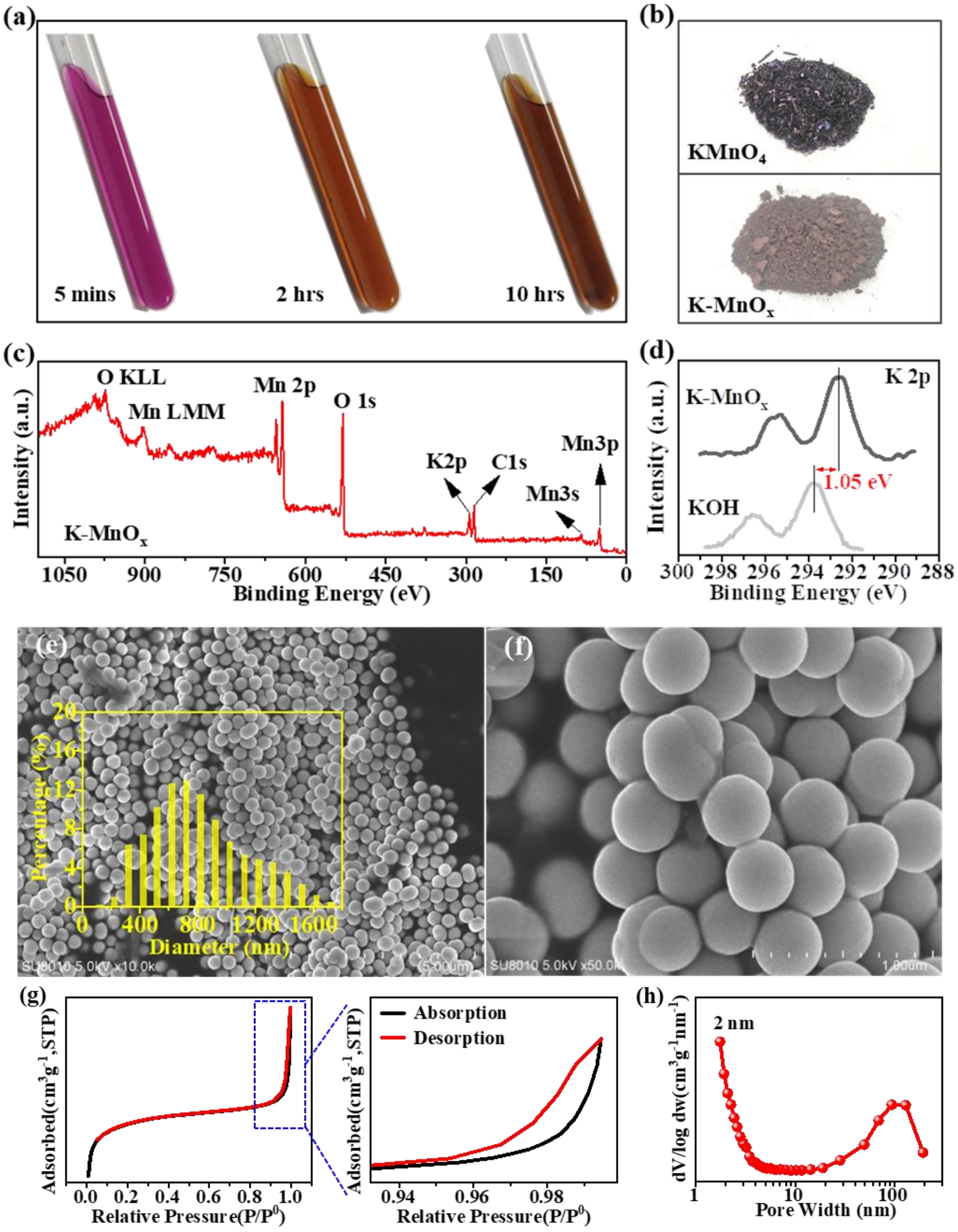


**Fig. S1.** (**a**) Color change of the $KMnO_4$-$NH_3$ $H_2O$ solution. The color changes from purple to brown gradually, suggesting the $Mn^{7+}$ being reduced by ammonium solution to $Mn^{3+}$ or $Mn^{4+}$. (**b**) Comparison of the $KMnO_4$ and K-$MnO_x$ samples. The noticeable difference in the colors of both samples indicates the disparity of the valence states of Mn in both samples. (**c**) XPS survey

spectrum of K-$MnO_x$. (**d**) K 2p spectra of K-$MnO_x$ and crystalline KOH. (**e,f**) SEM images of K-$MnO_x$ and the inset in figure **e** is its particle size distribution image. (**g**) $N_2$ adsorption/desorption isotherms and (**h**) pore size distribution curve of K-$MnO_x$.

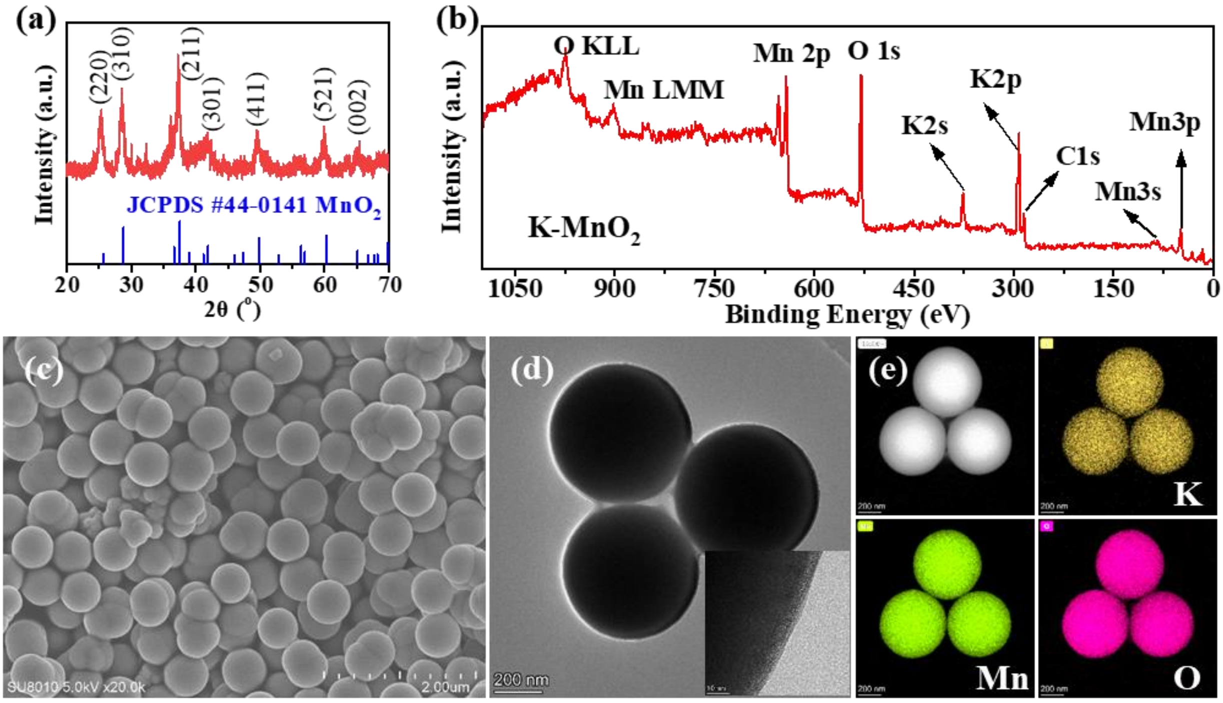


**Fig. S2.** (**a**) XRD pattern, (**b**) XPS survey spectrum, (**c**) SEM image, (**d**) TEM image, (**e**) TEM-HADDF-Mapping images of K-$MnO_2$. The strong diffraction peaks of K-$MnO_2$ agree well with the peaks of $MnO_2$ (JCPDS #44-0141), indicating the transformation of the amorphous phase into a crystalline phase. The XPS and TEM-Mapping tests detect a strong signal of K, and hence we named the annealed K-$MnO_x$ as the K-contained $MnO_2$ (K-$MnO_2$). The morphology of K-$MnO_2$ still retains microspheres like K-$MnO_x$. However, crystal water has been removed during the heating treatment process according to the TGA result shown in **Fig. 1c**.

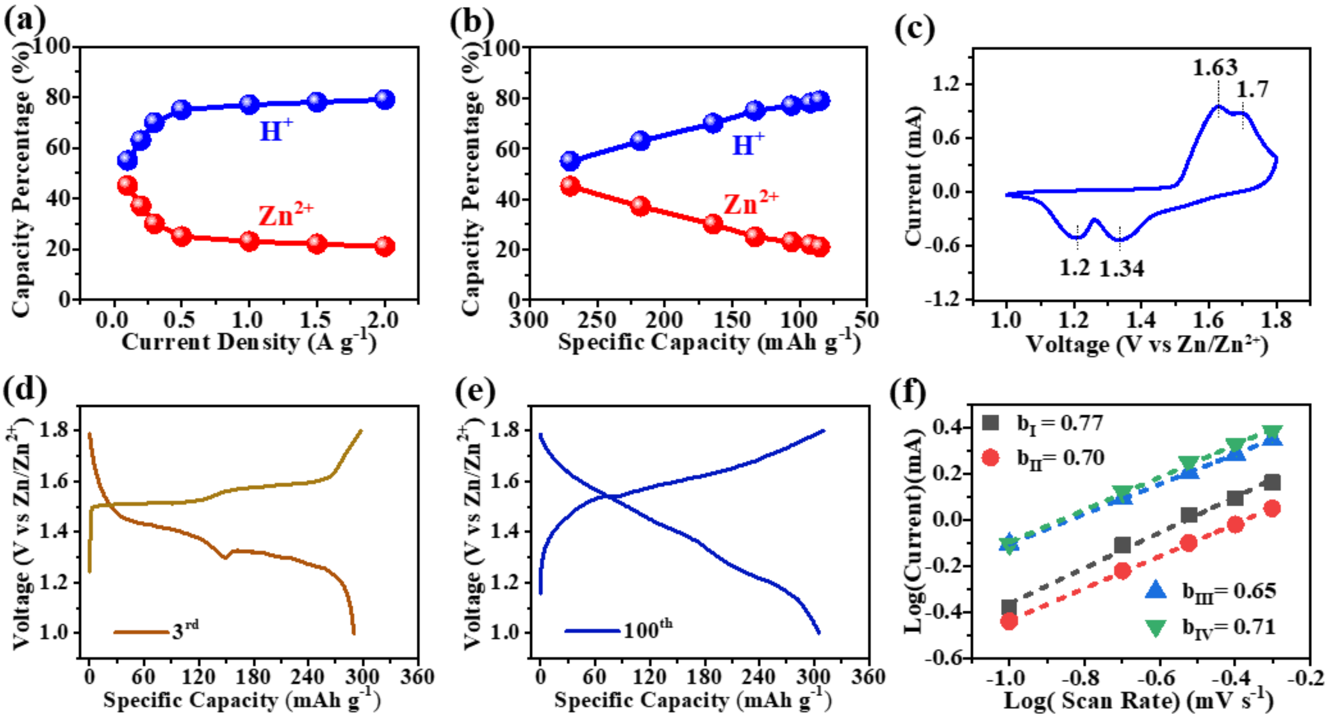


**Fig. S3.** The $Zn^{2+}$/$H^{+}$- capacity contribution percentage of K-$MnO_x$ (in K-$MnO_x$//Zn) at various (**a**) current density (A $g^{-1}$) and (**b**) specific capacity (mAh $g^{-1}$). (**c**) A typical CV curve of K-$MnO_x$ at 1.0 mV $s^{-1}$ after 100 discharge-charge cycles. GCD curves acquired after (**d**) 3 and (**e**) 100 cycles at 0.2 A $g^{-1}$. (**f**) The linear relationship of Log (scan rate) (mV $s^{-1}$) and Log (current) (mA).

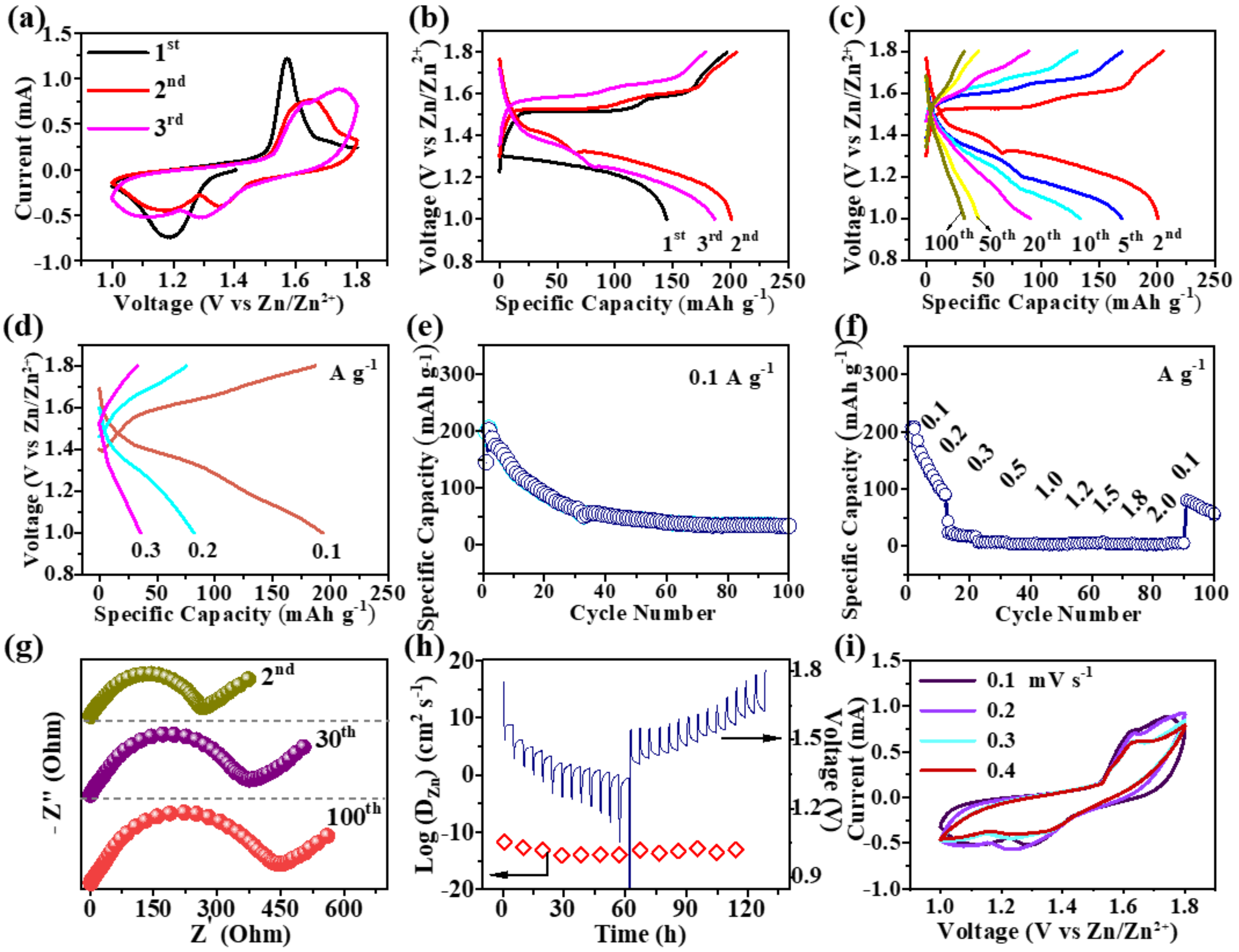


**Fig. S4.** (**a**) CV and (**b-d**) GCD curves of K-$MnO_2$. (**e**) Cycling and (**f**) rate performance of K-$MnO_2$ electrode. (**g**) Nyquist plots of K-$MnO_2$ electrode acquired after the 2$^{nd}$, 30$^{th}$, and 100$^{th}$ cycles. (**h**) GITT curves and corresponding $Zn^{2+}$ diffusion coefficient for K-$MnO_2$ electrode (acquired at 0.05 mA). (**i**) CV curves of K-$MnO_2$ electrode at various scan rates from 0.1 to 0.4 mV $s^{-1}$.

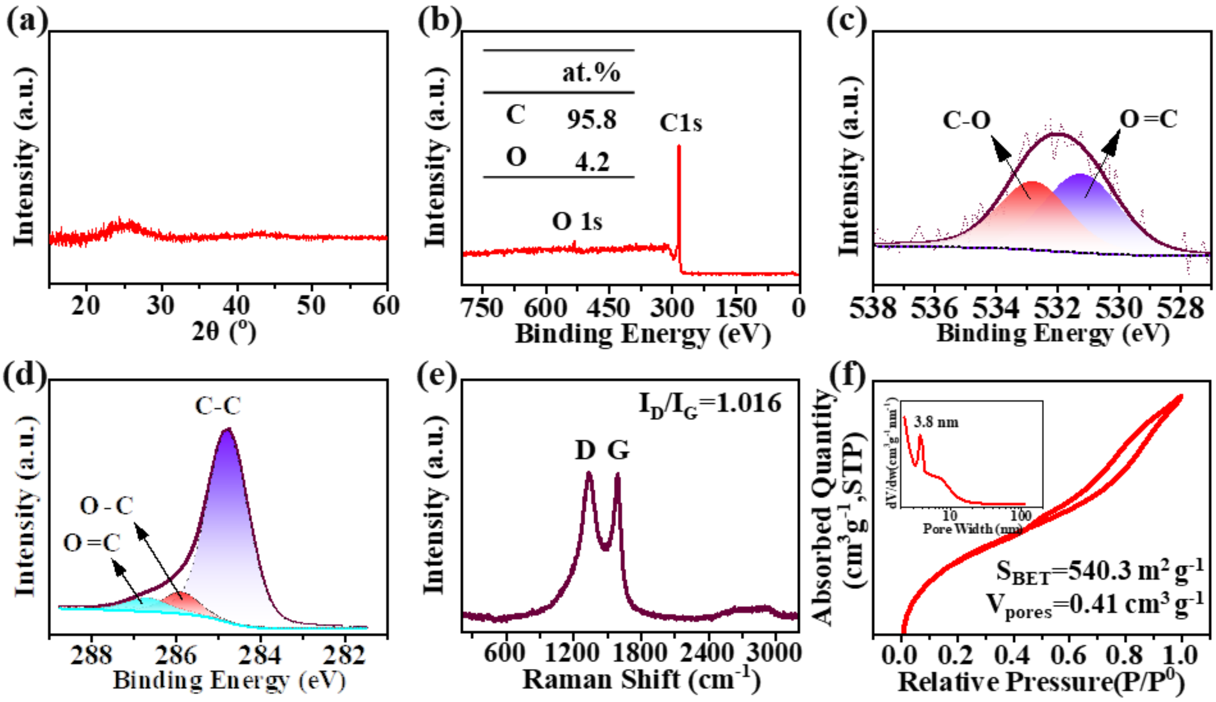


**Fig. S5.** (**a**) XRD pattern of AC. (**b**) XPS survey spectrum of AC and the inset is its atomic percentage of C and O. (**c,d**) Fitted XPS spectra of O 1s and C 1s. (**e**) Raman spectrum of AC. (**f**) $N_2$ adsorption-desorption and pore-size-distribution curves of AC.

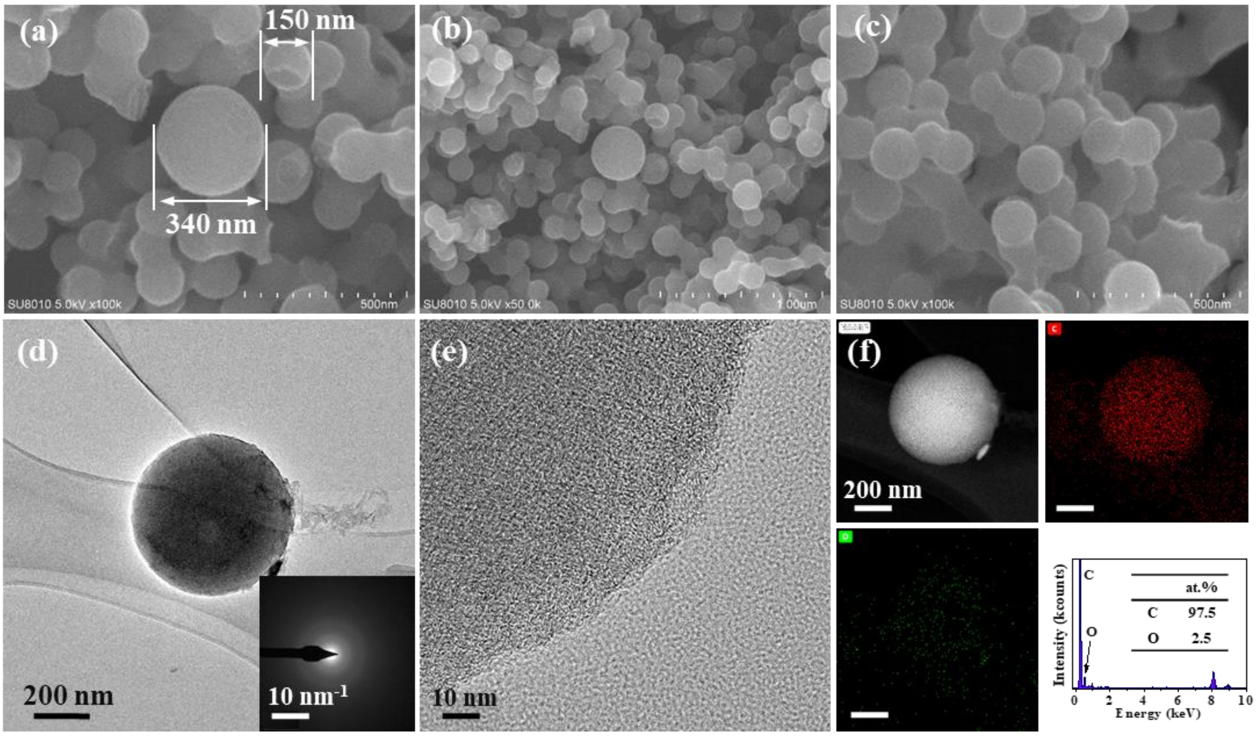


**Fig. S6.** (**a-c**) SEM and (**d-f**) TEM-SAED-EDS-Mapping images of AC.

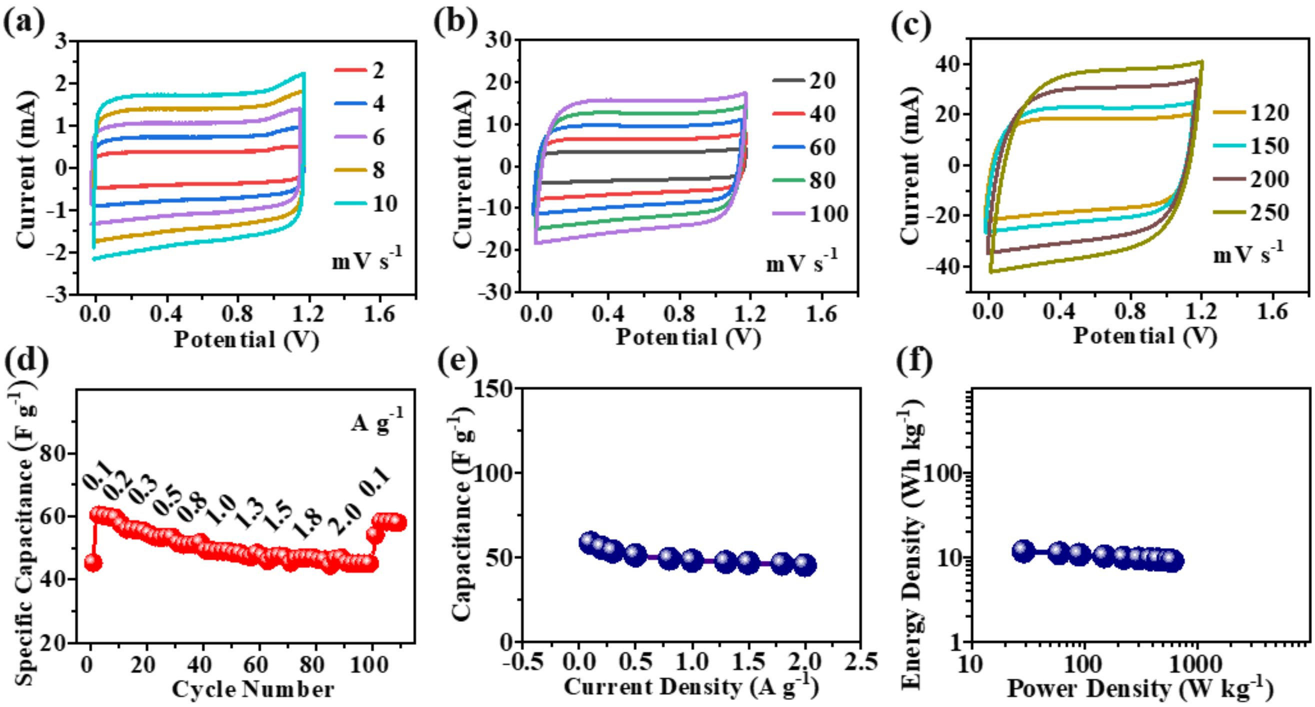


**Fig. S7.** Electrochemical performance of AC electrodes in AC//AC devices. (**a-c**) CV curves, and (**d**) Rate performance. (**e**) Specific capacitances at various current densities. (**f**) Ragone plot.

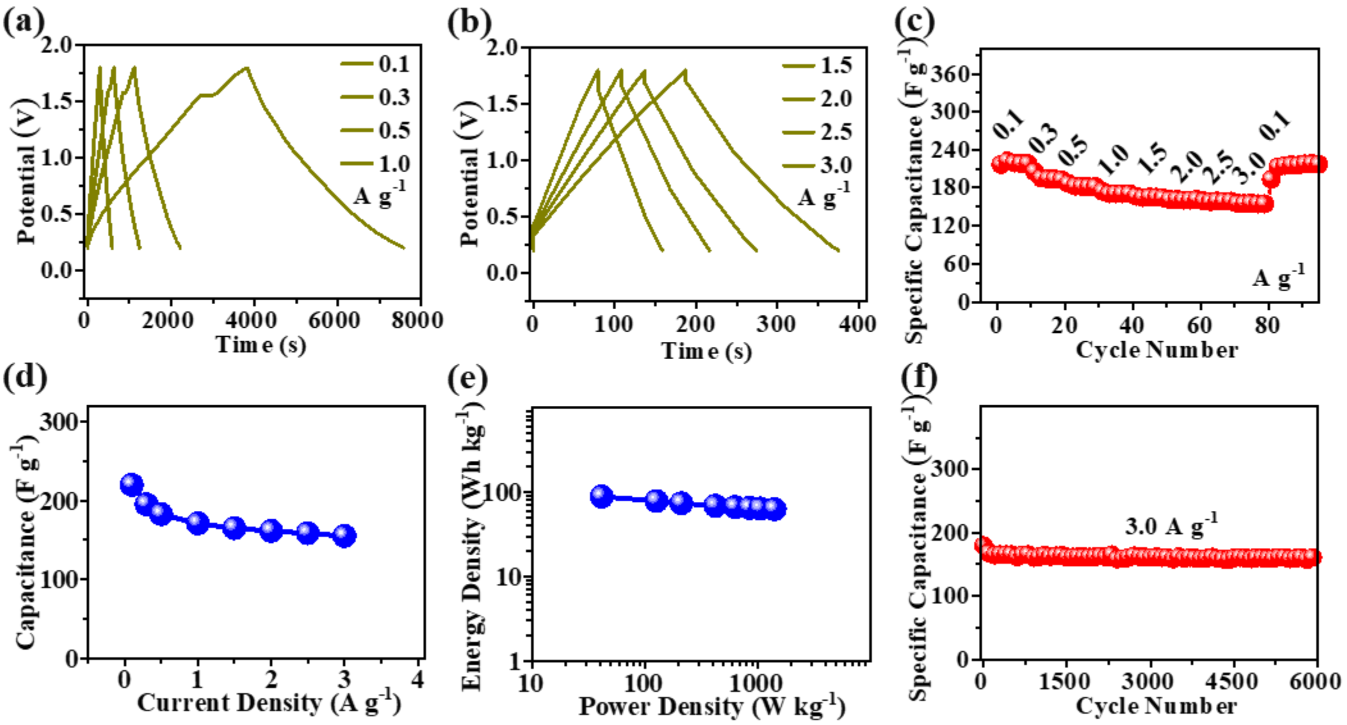


**Fig. S8.** Electrochemical performance of AC electrodes in AC//Zn devices. (**ab**) GCD curves, (**c**) Rate performance. (**d**) Specific capacitances at various current densities. (**e**) Ragone plot. (**f**) Long-cycling performance.

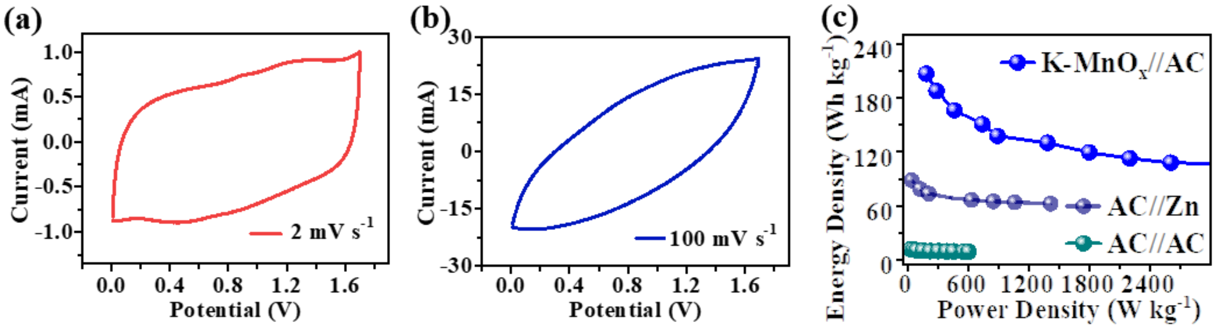


**Fig. S9.** CV curves of K-MnO$_x$ electrode in K-MnO$_x$//AC at (**a**) 2.0 mV s$^{-1}$ and (**b**) 100 mV s$^{-1}$. (**c**) Comparison of K-MnO$_x$//AC, AC//Zn, and AC//AC devices in Ragone plots.

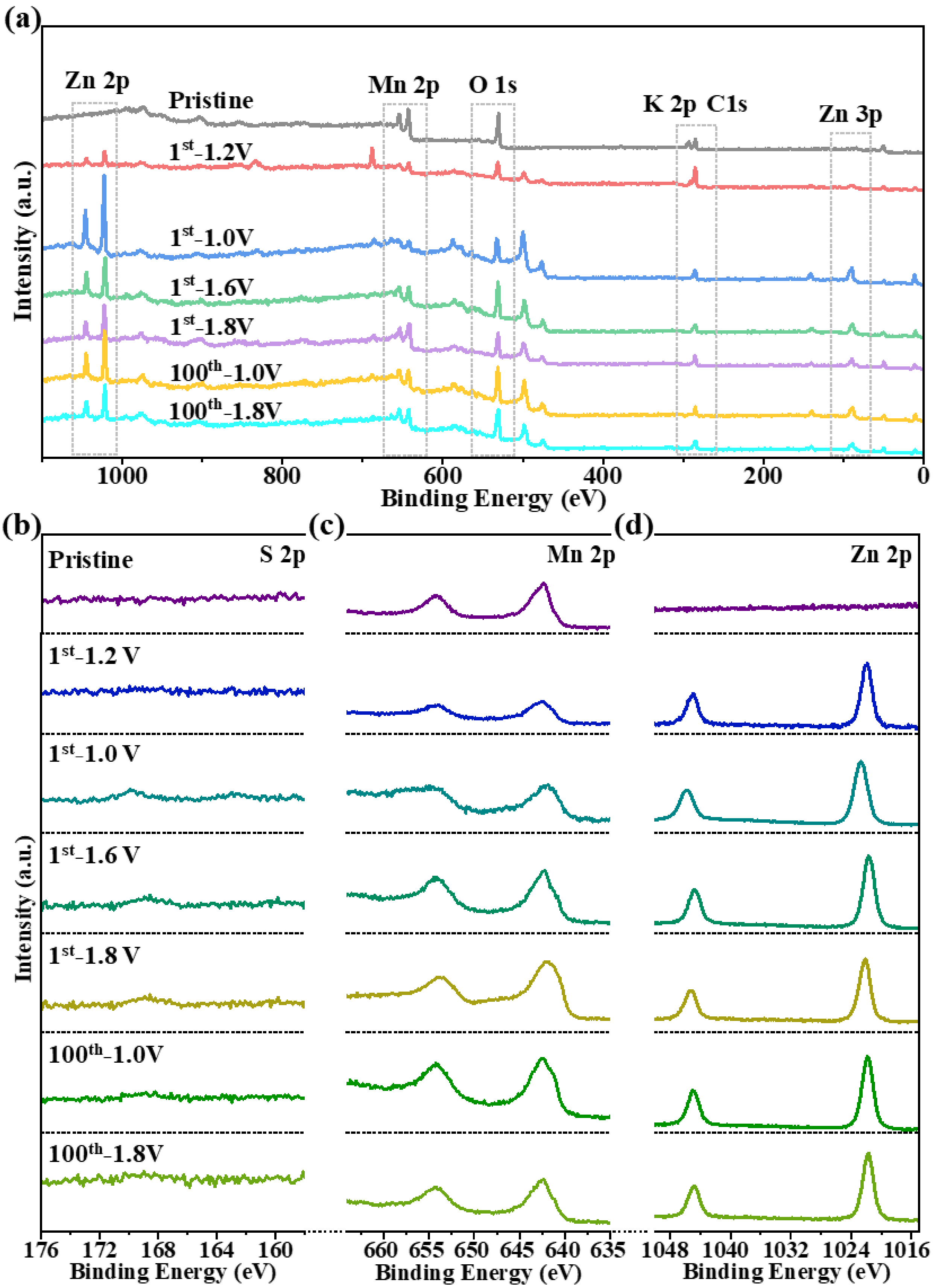


**Fig. S10.** XPS (**a**) survey, (**b**) S 2p, (**c**) Mn 2p, and (**d**) Zn 2p spectra of K-$MnO_x$ electrodes at various discharge-charge states.

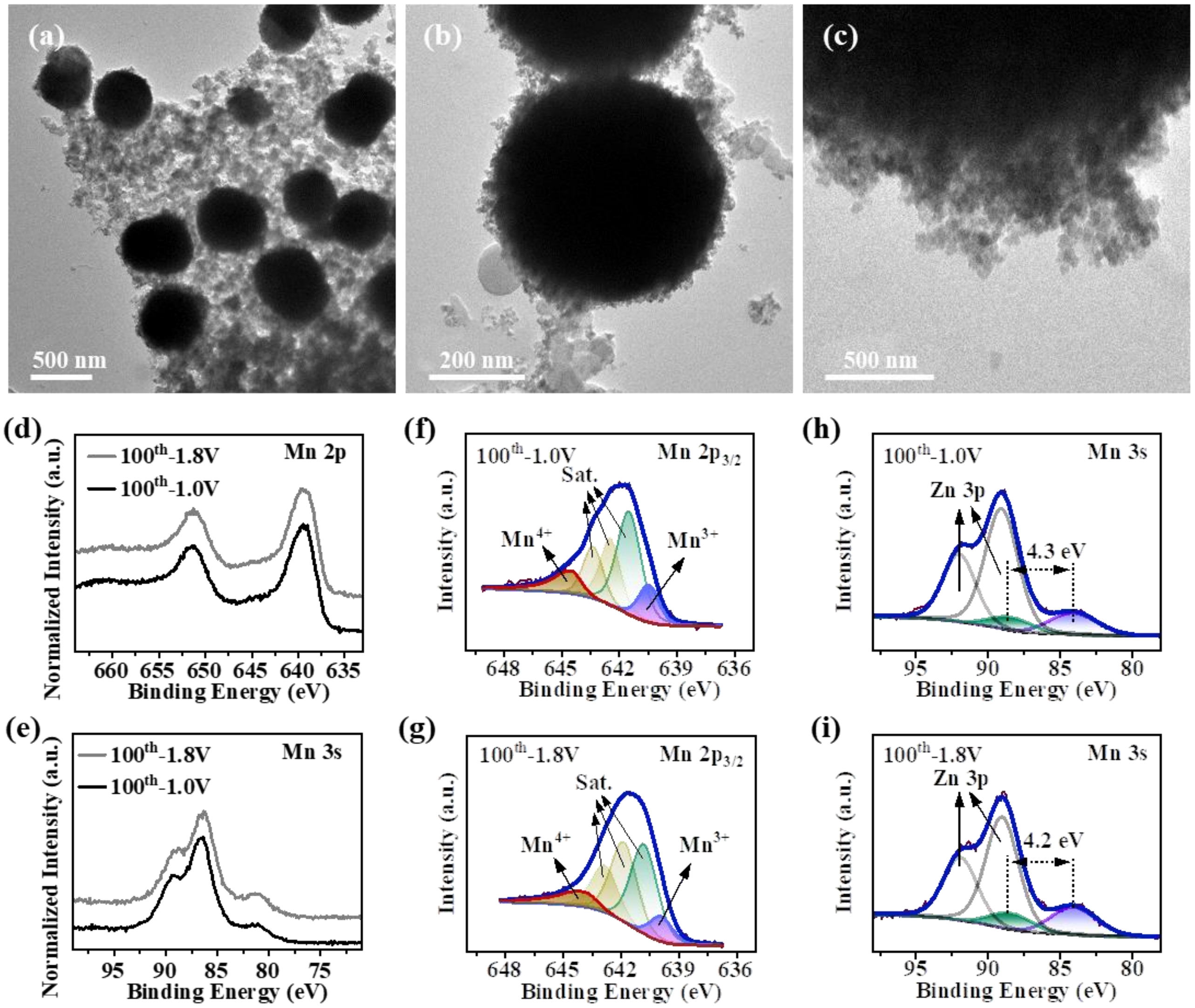


**Fig. S11.** (**a-c**) TEM images of K-$MnO_x$ electrode after 100 cycles at 0.2 A $g^{-1}$. In these images, we can observe the microspherical Zn-buserite composed of many nanoparticles. (**d,e**) Comparison of the Mn 2p and Mn 3s spectra of $100^{th}$-1.0 V and $100^{th}$-1.8 V K-$MnO_x$ electrodes. (**f-i**) The fitted Mn 2p and Mn 3s spectra of $100^{th}$-1.0 V and $100^{th}$-1.8 V K-$MnO_x$ electrodes.

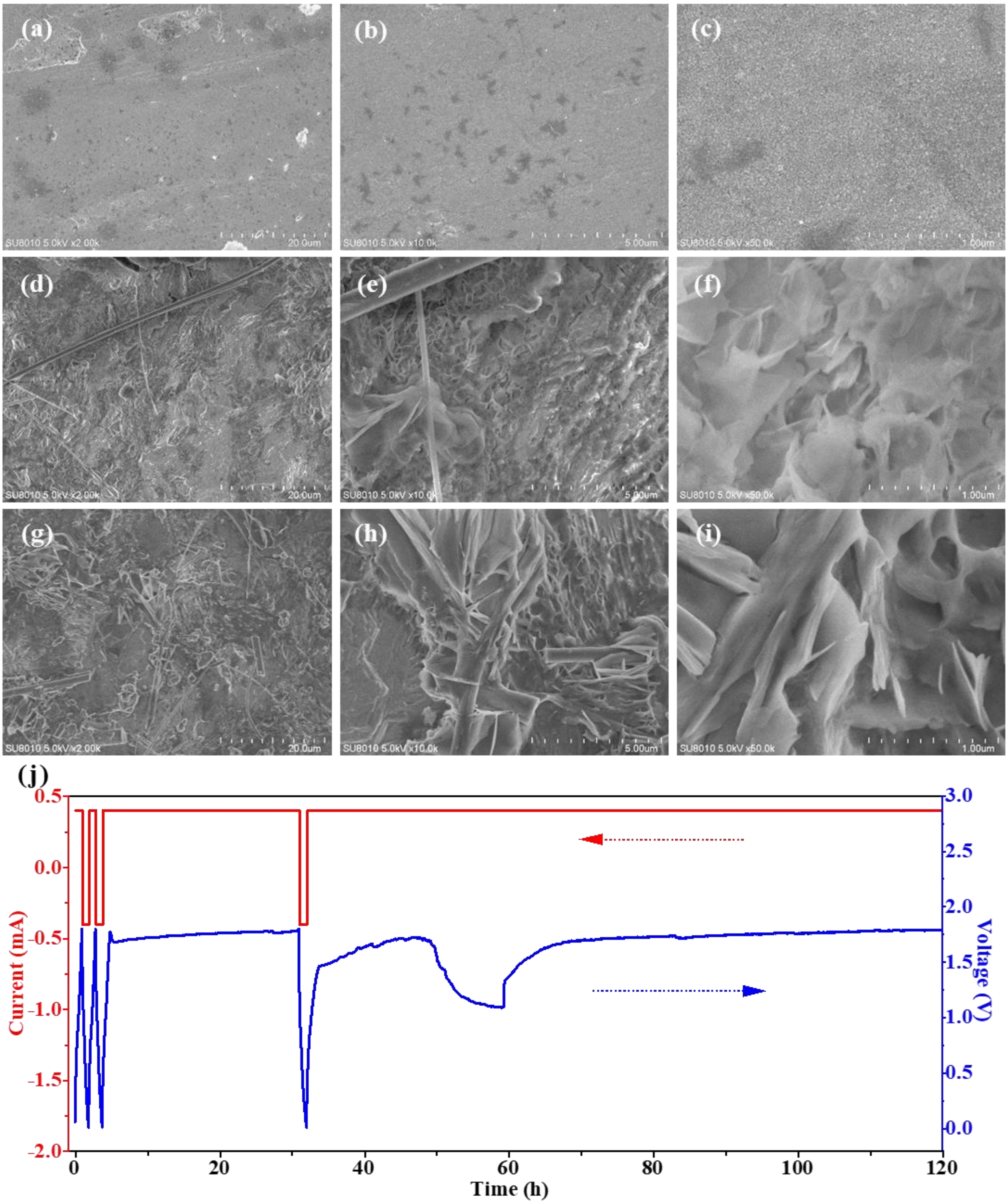


**Fig. S12.** SEM images of (**a-c**) pristine Zn foil anode, (**d-f**) Zn anode in K-$MnO_x$//Zn, and (**g-i**) Zn anode in AC//Zn devices. (**j**) Typical GCD curve obtained from a K-$MnO_x$//Zn device, exhibiting a short circuit caused by Zn-dendrites.

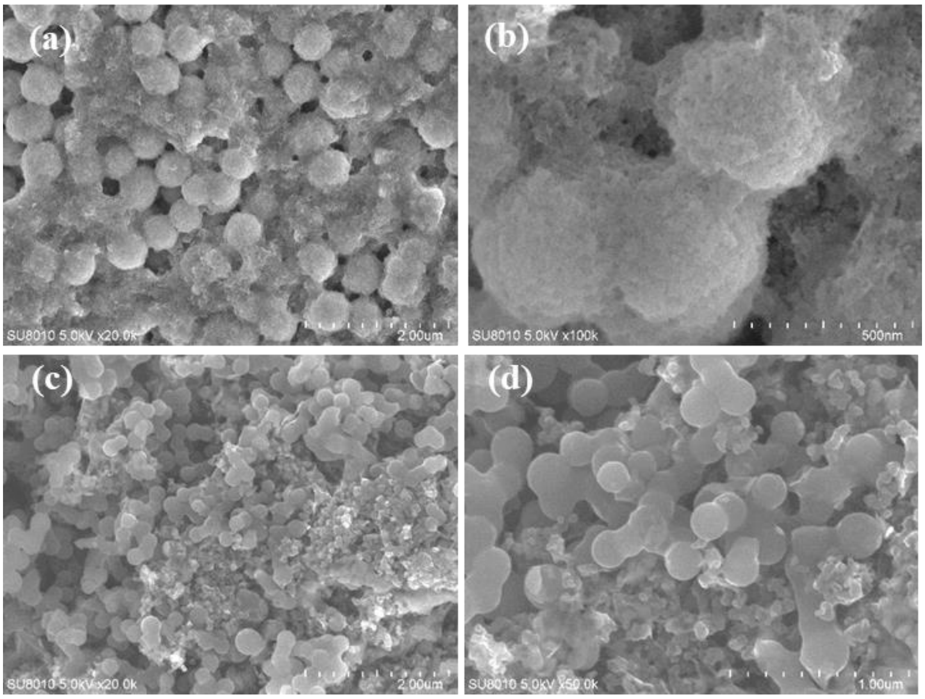


**Fig. S13.** SEM images of (**ab**) K-$MnO_x$ cathode and (**cd**) AC anode in a K-$MnO_x$//AC device.

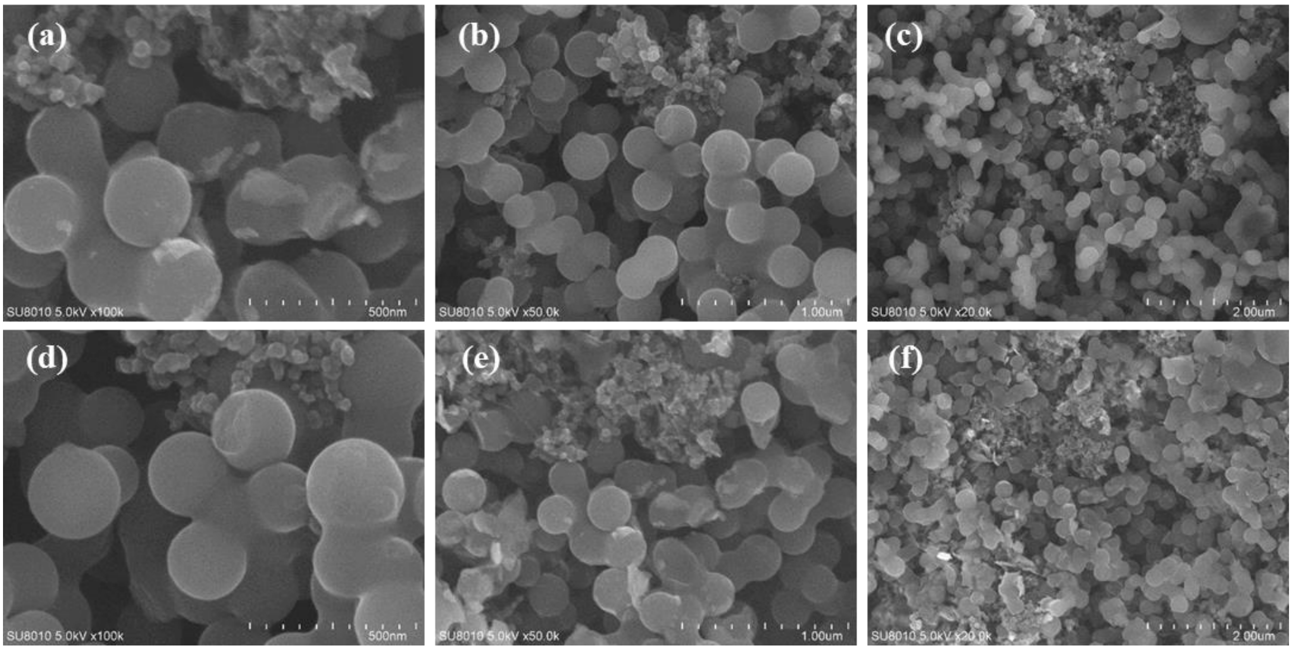


**Fig. S14.** SEM images of (**a-c**) pristine AC electrode, and (**d-f**) AC electrode in AC//Zn device after 100 cycles at 0.2 A $g^{-1}$.

**Table S1.** The atomic ratio of K-$MnO_x$ detected by XPS/ICP/TEM

| | **K: Mn: O** |
|---|---|
| **XPS** | **0.186: 1: 1.387** |
| **ICP** | **0.182: 1: -** |
| **TEM** | **0.185: 1: 1.278** |

**Table S2.** Comparison of electrochemical performance (including capacitance (**C**, F g$^{-1}$), energy density (**E**, Wh kg$^{-1}$), power density (**P**, kW kg$^{-1}$), and capacitance retention (**CR**, %) of our work with previous reports.

| Energy storage systems (cathode//electrolyte//anode) | Voltage ranges (V) | **C** (F g$^{-1}$) (at A g$^{-1}$) | **E** (Wh kg$^{-1}$) (at A g$^{-1}$) | **P** (kW kg$^{-1}$) (at A g$^{-1}$) | **CR** % (cycles) | Ref./Year |
|---|---|---|---|---|---|---|
| K-$MnO_x$//2M $Zn(CF_3SO_3)_2$ + 2M $Mn(CF_3SO_3)_2$//AC | 0.01-1.7 | 515.0 (0.15) | 206.7 (0.15) | 183.6 (0.15) | - | Our work |
| | | 254.1 (5.0) | 102.2 (5.0) | 3.06 (5.0) | 92.9% (20,000) | Our work |
| | | 116.0 (20.0) | 46.6 (20.0) | 16.9 (20.0) | - | Our work |
| Zn-$MnO_2$//$ZnSO_4$//AC | 0-2 | 282.9 (0.2) | 157.2 (0.2) | 16 (0.2) | 80.2% (30,000) | [5]/2022 |
| Zn-$MnO_2$//2M $ZnSO_4$ + 0.5M $MnSO_4$//AC | 0-2 | 343.1 (0.1) | 190.63 (0.1) | 20 (0.1) | - | [5]/2022 |
| Zn-$MnO_2$//$ZnCl_2$//AC | 0-2 | 167.6 (0.1) | 93.1 (0.1) | 20 (0.1) | - | [5]/2022 |
| a-MEGO//$Zn(CF_3SO_3)_2$//Zn | 0-1.9 | 212 | 106.3 | 31.4 | 93% (80,000) | [6]/2019 |
| $ZnMn_2O_4$-C //3M $Zn(CF_3SO_3)_2$//Zn | 0.8-1.9 | - | 202 | - | 94% (500) | [7]/2016 |
| PSC-A600//$Zn(CF_3SO_3)_2$//AC | 0.2-2.2 | 403 (0.2) | 147 | 15.7 | 92.2% (10,000) | [8]/2020 |
| AC//$Zn(CF_3SO_3)_2$//Zn | 0-1.8 | 170 (0.1) | 53 | 1.7 | 91% (20,000) | [9]/2018 |
| AC//$ZnSO_4$//Zn | 0.8-1.8 | 272 (0.1) | 84 | 14.9 | 91% (10,000) | [10]/2018 |
| δ-$MnO_2$//2M $ZnSO_4$ + 0.1 M $MnSO_4$//MXene | 0-1.9 | - | 90 | 3.8 | 80.7% (16,000) | [11]/2019 |
| ZIC-CC/ZIF-8 | 0.2-1.8 | 302/188 (0.5/20) | 107.4 | 16.2 | 100% (16,000) | [12]/2021 |
| EG/PANI//2M $ZnSO_4$//POP-TAPP-NTCA | 0.1-1.5 | 172 (0.13) | 48 | 85 | 90% (1,100) | [13]/2021 |
| sn-$MnO_2$//1M $Na_2SO_4$//sn-$MnO_2$ | 0.01-1.0 | 868 (3.0) | - | - | 91% (10,000) | [14]/2015 |
| $MnO_2$-CNTs//2M $ZnSO_4$ + 0.1M $MnSO_4$//MXene | 0.01-1.9 | 115.1 | 98.6 | 2.48 | 83.6% (15,000) | [15]/2019 |
| OFCNT-5//PAM-$ZnSO_4$//Zn | 0.2-1.8 | 1556.8 mF cm$^{-2}$ | 553.5μW h cm$^{-2}$ | 26.83 mW cm$^{-2}$ | 91.8% (90,000) | [16]/2022 |

| | | | | | | |
|---|---|---|---|---|---|---|
| HPCS-900//$ZnSO_4$//Zn | 0.1-1.7 | 104.7 mAh $g^{-1}$ | 90.17 | 0.0812 | 95.24% (30,000) | [17]/2022 |
| BSG//$Zn(CF_3SO_3)_2$//Zn | 0.1-1.8 | 224 (0.5) | - | - | 52.2% ($20Ag^{-1}$) | [18]/2022 |
| NPFC//$Zn(CF_3SO_3)_2$(aq.)//Zn | 0.1-1.8 | 163.6 mAh $g^{-1}$ | 85.7 | 35.9 | 97.4% (20,000) | [19]/2022 |
| rGO-200//$ZnSO_4$//Zn | 0.01-1.8 | 245 (0.5) | 266μWh $cm^{-2}$ | - | 75% (10,000) | [20]/2022 |
| GCNF//$Zn(CF_3SO_3)_2$//Zn | 0.2-1.8 | 173.8 mAh $g^{-1}$ | 113.2 | 206.2 | 93.5% (10,000) | [21]/2022 |
| $NbMo_8S_6$/NC//$ZnClO_4$+$NaClO_4$//Zn | 0-1.5 | 167.89 mAh $g^{-1}$ | 188.87 | 2.5 | 87.60%. (15,000) | [22]/2023 |
| N-OPCNF//$ZnSO_4$//Zn | 0.2-1.8 | 136 mAh $g^{-1}$ | 98.28 | 33.2 | 99.2% (200,000) | [23]/2022 |
| PPy/rGO//$Zn(CF_3SO_3)_2$//Zn | 0.2-1.6 | 92.5 mF $cm^{-2}$ | 25.2mW h $cm^{-2}$ | - | 92% (10,000) | [24]/2022 |
| 3D MGC//$ZnSO_4$+$NaClO_4$//Zn | 0-2 | 315 F $g^{-1}$ (30mV $s^{-1}$) | 203 | 4.9 | 96.75% (30,000) | [25]/2022 |
| $Ti_3C_2T_x$//$ZnSO_4$//Zn | - | 323.7 | 20.8 mWh $cm^{-3}$ | 249.9 μWh $cm^{-2}$ | 92.7% (10,000) | [26]/2022 |
| LNPC//$ZnSO_4$//Zn | 0.2-1.8 | 266 | - | - | - | [27]/2022 |
| rGO/NbPO//$ZnSO_4$+$NaClO_4$//Zn | 0.15-1.6 | 191.88 | 56.03 | - | 76.81% (50,000) | [28]/2022 |
| $B_2S_3C$//$ZnSO_4$//Zn | 0.2-1.8 | 182.6 (0.1) | 292.2 | 0.0622 | 96.2% (10,000) | [29]/2022 |
| AC-10//$ZnSO_4$//Zn | 0.2-1.8 | 160 (1.0) | - | - | 92.1% (10,000) | [30]/2022 |
| AC-CNT//$ZnSO_4$//Zn | 0.2-1.8 | 4.259 F $cm^{-2}$ | 1514μW h $cm^{-2}$ | - | ~100% (10,000) | [31]/2022 |
| WAPC-x//$ZnSO_4$//Zn | 0.3-1.9 | 158.9 mAh $g^{-1}$ | 127.1 | - | 88.2% (15,000) | [32]/2022 |
| NPCNs//$ZnSO_4$//Zn | 0.15-1.7 | 173.9 mAh $g^{-1}$ (0.2 A $g^{-1}$) | 64.8 | 1.099 | 118.2% (15,000) | [33]/2022 |
| C-X//$ZnSO_4$//Zn | 0.2-1.8 | 121.7 mAh $g^{-1}$ | 109.3 | 15.6 | ~100% (30,000) | [34]/2022 |

## References

[1] Z. Wang, B. Zeng, D. Zhou, L. Tai, X. Liu, W. Lau, *Chem. Eng. J.* **2022**, *428*, 132637.

[2] Y. Cai, R. Chua, S. Huang, H. Ren, M. Srinivasan, *Chem. Eng. J.* **2020**, *396*, 125221.

[3] B. Lee, H. R. Lee, H. Kim, K. Y. Chung, B. W. Cho, S. H. Oh, *Chem. Commun.* **2015**, *51* (45), 9265.

[4] Y. Jiang, D. Ba, Y. Li, J. Liu, *Adv. Sci.* **2020**, *7* (6), 1902795.

[5] S. He, Z. Mo, C. Shuai, W. Liu, R. Yue, G. Liu, H. Pei, Y. Chen, N. Liu, R. Guo, *Appl. Surf. Sci.* **2022**, *577*, 151904.

[6] S. Wu, Y. Chen, T. Jiao, J. Zhou, J. Cheng, B. Liu, S. Yang, K. Zhang, W. Zhang, *Adv. Energy Mater.* **2019**, *9* (47), 1902915.

[7] N. Zhang, F. Cheng, Y. Liu, Q. Zhao, K. Lei, C. Chen, X. Liu, J. Chen, *J. Am. Chem. Soc.* **2016**, *138* (39), 12894.

[8] Z. Li, D. Chen, Y. An, C. Chen, L. Wu, Z. Chen, Y. Sun, X. Zhang, *Energy Storage Mater.* **2020**, *28*, 307.

[9] H. Wang, M. Wang, Y. Tang, *Energy Storage Mater.* **2018**, *13*, 1.

[10] L. Dong, X. Ma, Y. Li, L. Zhao, W. Liu, J. Cheng, C. Xu, B. Li, Q. Yang, F. Kang, *Energy Storage Mater.* **2018**, *13*, 96.

[11] J. Shi, S. Wang, Q. Wang, X. Chen, X. Du, M. Wang, Y. Zhao, C. Dong, L. Ruan, W. Zeng, *J. Power Sources* **2020**, *446*, 227345.

[12] C. Leng, Z. Zhao, J. Guo, R. Li, X. Wang, J. Xiao, Y. V. Fedoseeva, L. G. Bulusheva, J. Qiu, *Chem. Commun.* **2021**, *57* (70), 8778.

[13] F. Cui, Z. Liu, D. Ma, L. Liu, T. Huang, P. Zhang, D. Tan, F. Wang, G. Jiang, Y. Wu, *Chem. Eng. J.* **2021**, *405*, 127038.

[14] Z. Liu, K. Xu, H. Sun, S. Yin, *Small* **2015**, *11* (18), 2182.

[15] S. Wang, Q. Wang, W. Zeng, M. Wang, L. Ruan, Y. Ma, *Nano Micro Lett.* **2019**, *11* (1), 70.

[16] Q. Chen, L. Cao, H. Wang, Z. Zhou, P. Xiao, *J. Power Sources* **2022**, *538*, 231586.

[17] K. Shang, Y. Liu, P. Cai, K. Li, Z. Wen, *J. Mater. Chem. A* **2022**, *10* (12), 6489.

[18] L. Zhao, W. Jian, J. Zhu, X. Zhang, F. Wen, X. Fei, L. Chen, S. Huang, J. Yin, N. R. Chodankar,

X. Qiu, W. Zhang, *ACS Appl. Mater. Interfaces* **2022**, *14* (38), 43431.

[19] F. Wei, Y. Wei, J. Wang, M. Han, Y. Lv, *Chem. Eng. J.* **2022**, *450*, 137919.

[20] H. Xu, W. He, Z. Li, J. Chi, J. Jiang, K. Huang, S. Li, G. Sun, H. Dou, X. Zhang, *Adv. Funct. Mater.* **2022**, *32* (16), 2111131.

[21] J. Gao, F. Guo, C. Ji, X. He, H. Mi, J. Qiu, *J. Mater. Chem. A* **2022**, *10* (46), 24639.

[22] S. J. Patil, N. R. Chodankar, S.-K. Hwang, P. A. Shinde, G. S. Rama Raju, K. S. Ranjith, S. V. Karekar, Y.-S. Huh, Y.-K. Han, *J. Mater. Chem. A* **2023**, https://doi.org/10.1039/D2TA07524E.

[23] H. He, J. Lian, C. Chen, Q. Xiong, C. C. Li, M. Zhang, *Nano Micro Lett.* **2022**, *14* (1), 106.

[24] X. Tian, H. Ma, Y. Gao, B. Xu, *J. Mater. Chem. A* **2022**, *10* (26), 14011.

[25] N. R. Chodankar, S. J. Patil, S. Lee, J. Lee, S.-K. Hwang, P. A. Shinde, I. V. Bagal, S. V. Karekar, G. S. R. Raju, K. S. Ranjith, D. P. Dubal, Y.-S. Huh, Y.-K. Han, *InfoMat* **2022**, *4* (10), e12344.

[26] J. Chen, M. Chen, W. Zhou, X. Xu, B. Liu, W. Zhang, C. Wong, *ACS Nano* **2022**, *16* (2), 2461.

[27] W. Zhang, J. Yin, W. Jian, Y. Wu, L. Chen, M. Sun, U. Schwingenschlogl, X. Qiu, H. N. Alshareef, *Nano Energy* **2022**, *103*, 107827.

[28] S. J. Patil, N. R. Chodankar, S.-K. Hwang, G. S. R. Raju, K. S. Ranjith, Y. Suk Huh, Y.-K. Han, *Energy Storage Mater.* **2022**, *45*, 1040.

[29] X. Zhang, Y. Zhang, J. Qian, Y. Zhang, L. Sun, Q. Wang, *Nanoscale* **2022**, *14* (5), 2004.

[30] J. Wu, R. Liu, M. Li, X. Luo, W. Lai, X. Zhang, D. Li, F. Yu, Y. Chen, *J. Energy Storage* **2022**, *48*, 103996.

[31] Y. Liu, S. Zheng, J. Ma, X. Wang, L. Zhang, P. Das, K. Wang, Z.-S. Wu, *Adv. Energy Mater.* **2022**, *12* (27), 2200341.

[32] H. Sun, C. Liu, D. Guo, S. Liang, W. Xie, S. Liu, Z. Li, *RSC Adv.* **2022**, *12* (38), 24724.

[33] P. Shang, M. Liu, Y. Mei, Y. Liu, L. Wu, Y. Dong, Z. Zhao, J. Qiu, *Small* **2022**, *18* (16), e2108057.

[34] F. Wei, H. Tian, P. Chen, Y. Lv, J. Huang, *Appl. Surf. Sci.* **2023**, *613*, 156021.
